# Balancing key stakeholder priorities and ethical principles to design a trial comparing intervention or expectant management for early-onset selective fetal growth restriction in monochorionic twin pregnancy: FERN qualitative study

**DOI:** 10.1136/bmjopen-2023-080488

**Published:** 2024-08-09

**Authors:** Tracy Karen Mitchell, Mariana Popa, Richard Edmund Ashcroft, Smriti Prasad, Andrew Sharp, Christine Carnforth, Mark Turner, Asma Khalil, Natasha Fenwick, Shauna Leven, The FERN study team, Kerry Woolfall

**Affiliations:** 1 Public Health, Policy and Systems, University of Liverpool, Liverpool, UK; 2 School of Law, City University of London, London, UK; 3 Fetal Medicine Unit, St George's University Hospitals NHS Foundation Trust, London, UK; 4 Vascular Biology Research Centre, Molecular and Clinical Sciences Research Institute, St George's University of London, London, UK; 5 Department of Women’s and Children’s Health, University of Liverpool, Liverpool, UK; 6 Clinical Directorate Professional Services, University of Liverpool, Liverpool, UK; 7 Molecular and Clinical Sciences Research Institute, St George's University of London, London, UK; 8 Research and Resources Officer, Twins Trust, London, Hampshire, UK; 9 Twins Trust, Woking, Surrey, UK

**Keywords:** obstetrics, qualitative research, medical ethics, surgery, fetal medicine, prenatal diagnosis

## Abstract

**Abstract:**

**Objectives:**

As part of the FERN feasibility study, this qualitative research aimed to explore parents’ and clinicians’ views on the acceptability, feasibility and design of a randomised controlled trial (RCT) of active intervention versus expectant management in monochorionic (MC) diamniotic twin pregnancies with early-onset (prior to 24 weeks) selective fetal growth restriction (sFGR). Interventions could include laser treatment or selective termination which could lead to the death or serious disability of one or both twins.

**Design:**

Qualitative semi-structured interviews with parents and clinicians. Data were analysed using reflexive thematic analysis and considered against the Principles of Biomedical Ethics.

**Participants and setting:**

We interviewed 19 UK parents experiencing (six mothers, two partners) or had recently experienced (eight mothers, three partners) early-onset sFGR in MC twin pregnancy and 14 specialist clinicians from the UK and Europe.

**Results:**

Participants viewed the proposed RCT as ‘ethically murky’ because they believed that the management of sFGR in MC twin pregnancy should be individualised according to the type and severity of sFGR. Clinicians prioritised the gestational age, size, decrease in growth velocity, access to the placental vessels and acceptability of intervention for parents. Discussions and decision-making about selective termination appeared to cause long-term harm (maleficence). The most important outcome for parents and clinicians was ‘live birth’. For clinicians, this was the live birth of at least one twin. For parents, this meant the live birth of both twins, even if this meant that their babies had neurodevelopmental impairment or disabilities.

**Conclusions:**

All three pregnancy management approaches for sFGR in MC twin pregnancy carry risks and benefits, and the ultimate goal for parents is to receive individualised care to achieve the best possible outcome for both twins. An RCT was not acceptable to parents or clinicians or seen as ethically appropriate. Alternative study designs should be considered to answer this important research question.

**STRENGTHS AND LIMITATIONS OF THIS STUDY**

- This study provides in-depth insight into the experiences of families who had different outcomes, including bereavement, resulting from their selective fetal growth restriction (sFGR) complicated monochorionic twin pregnancy, as well as specialist clinicians managing sFGR pregnancies.
- Data analysis was informed by the biomedical ethical principles which provided insight into the challenging ethics of running the proposed study in a randomised fashion.
- Parents had experience of being offered the pregnancy management options that are proposed for the randomised controlled trial due to being recruited via hospital sites (currently pregnant) and social media (pregnant within the last 3 years).
- An ethicist was involved in the analysis of findings.
- Limited to participants who could speak English.

## Introduction

 Around a third of twin pregnancies share a placenta (monochorionic (MC) twins);^1^ this poses unique difficulties for pregnancy management including selective fetal growth restriction (sFGR) where one twin grows significantly slower than the other. sFGR affects between 10% and 15% of MC twin pregnancies.^2^ Despite advances in antenatal care, sFGR in MC twin pregnancy is associated with preterm birth, stillbirth, neonatal death^3^,^5^ and neurodisability, including cerebral palsy.^4 6 7^ Early-onset sFGR, occurring before 24 weeks gestation, is less common but poses greater risk to the fetus and substantial management difficulties due to the distance from viability and the need to account for the welfare of both twins.^5^ One study investigating 119 pregnancies (n=75/63% early onset sFGR and n=44/37% late onset sFGR) showed that in early-onset sFGR, survival of one (n=62/82.7%) or both twins (n=55/73.3%) were lower compared with late-onset sFGR (one twin n=42/95.5%; both twins n=39/88.6%).^8^

There is a lack of high-quality evidence on the best way to manage sFGR in twin pregnancies, leading to uncertainty among clinicians about clinical management and how best to discuss options with parents to help them make difficult decisions about management. Depending on where parents live and which clinician they see,^4^ the three options offered in the UK are expectant management, selective termination of the sFGR twin and laser treatment (see table 1).

**Table 1:** Management options offered in the UK to women with a monochorionic twin pregnancy complicated by selective fetal growth restriction (sFGR)

Expectant management	Involves close monitoring of the twins. Expectant management aims to balance the risks of continuing the pregnancy and prematurity against the risk of intrauterine demise of the sFGR twin, which can then lead to the death of, or neurological damage in, the larger twin.
Selective termination of the sFGR twin	A procedure with bipolar cord coagulation or radiofrequency ablation or selective laser photocoagulation to block the blood flow through the umbilical cord from the placenta to the smaller twin. The sFGR twin dies which allows the larger twin to continue growing and gain maturity, hopefully delivering at a normal gestation. This procedure may also protect the larger twin from death or neurological damage.
Fetoscopic laser treatment	Placental laser photocoagulation to close the connections between the babies in the placenta with the aim of balancing the blood supply to both babies. This is likely to be a complex surgery and may worsen outcomes for the sFGR twin.

sFGRselective fetal growth restriction

A randomised control trial (RCT) could provide clinicians and parents with evidence to inform decisions about the management option that would have the most favourable outcome for MC twin pregnancies with early-onset sFGR. There are, however, many challenges for a potential RCT in this situation, including a low incidence of the condition, uncertainty about clinician equipoise, parents’ information needs and preferences and whether it is ethically acceptable to randomise women to expectant management or active intervention, which may lead to serious disability or the death of one or both twins.

## Methods

### Study design

The FERN study^9^ involved three work packages, including (1) prospective UK multicentre observational study, (2) qualitative study, (3) international survey^10^ and consensus meeting. This paper presents the findings of the qualitative work package 2 phase of the study. The aim of work package 2 was to explore parents’ and clinicians’ perspectives on how the future clinical trial should be designed (including recruitment and consent approaches and the design of research materials), the factors that influence parents’ and clinicians’ decision-making and the acceptability of a future clinical trial.

Following ethics approval (REC reference: 20/SW/0156), we conducted online or telephone interviews with English-speaking women and their partners (where applicable) in the UK who were experiencing early-onset sFGR in MC twin pregnancy (or with experience in the last 3 years), and English-speaking clinical staff involved in the management of MC twin pregnancies in the UK and Europe, to explore their views on the feasibility, acceptability and design of a proposed RCT, with one ‘watch and wait’ expectant management arm, and two intervention arms: (1) selective termination of the sFGR twin and (2) laser treatment, for early-onset sFGR in MC twin pregnancy. Interviews were conducted between September 2022 and March 2023.

We used previous research^11 12^ to develop the parent, partner and clinician participant information sheets (PIS) (see online supplemental files 1-3), while ongoing study findings were used to develop parent and clinician interview topic guides (see online supplemental files 4-5) as part of an iterative process. Interview topic guides included questions on the experience of management of MC twin pregnancies that were complicated by sFGR, decision-making processes, proposed trial design, information materials, trial acceptability, willingness to randomise/be randomised, prioritised outcomes and clinician training needs. The consolidated criteria for reporting qualitative research (COREQ) checklist^13^ was used to aid reporting (see online supplemental file 6).

### Patient and Public Involvement and Engagement (PPIE)

Our PPIE members include six coapplicants: Michelle Watson, Jessica Mendoza, Danielle Harding and Joel Marsden (two with personal experience of sFGR in MC twin pregnancy) and Natasha Fenwick and Shauna Leven from Twins Trust (https://twinstrust.org), which is a registered charity who support parents through every milestone of their journey with twins, triplets or more. The PPIE members were involved in the grant development, design, recruitment for, conduct, progress and/or findings of the FERN study; and/or as members of the study oversight/steering committee; and/or attended the work package 3 Key Stakeholder meeting in London (3 July 2023) and/or reviewing and providing input on draft research information materials for this qualitative study and reviewing drafts of this manuscript.

### Recruitment and sampling procedure

Based on previous qualitative feasibility studies,^11 14^ we anticipated that we would need to interview 15–25 parents and clinicians to reach information power,^15^ which is the point at which data address the study aims; sample variance^13^ (eg, parents offered expectant management or intervention, bereaved and non-bereaved parents and clinicians in favour of intervention and expectant management); our reflexive and interpretive approach to theory and analysis^16 17^ and sufficient quality of interview dialogue.^15^ We planned to hold additional focus groups if divergence in opinion was observed in interview data, but these were not required. Parents were recruited via work package 1 hospital sites and social media (Facebook and Twitter, with the support of Twins Trust).

### Eligibility screening and conduct

Research midwives at hospitals (n*=*5/17) involved in work package 1 checked eligibility and approached parents with FERN study information, which included details of the qualitative study. MP (female, Social Scientist) or TKM (female, Social Scientist) contacted parents who had registered interest to participate in an interview to arrange a convenient time for an online or telephone interview (according to their preference). For social media recruitment, MP and TKM responded to parents’ expressions of interest to take part in an interview in sequential order. Once eligibility had been confirmed, parents were emailed a copy of the Parent PIS which explained what would happen during their interview (see online supplemental file 7). Once parents confirmed their continued interest, they were then sent a proposed trial PIS and the core outcome measures list (see online supplemental file 8), derived from a review of the literature and Core Outcome set for this population.^18^

TKM and MP contacted work package 1 site clinicians and attendees at the International Society of Ultrasound in Obstetrics and Gynaecology World Congress 2022 to invite them to take part in an interview. Clinicians who expressed an interest in taking part were sent the Practitioner PIS, proposed Inclusion and Exclusion Criteria (see online supplemental file 9), and the same outcome measures list that was sent to parents before interview.

TKM and MP facilitated parent and clinician interviews using the topic guides.^19^ Interviews stopped when information power^15^ was reached. Parents then received a £30 Amazon voucher via email to compensate them for their time.

### Analysis

MP and TKM conducted the analysis with oversight from KW (female, Social Scientist) and RA (male, Ethicist). Digital audio recordings of interviews were transcribed verbatim by a professional transcription company (UK Transcription, Brighton, UK). Transcripts were checked for accuracy and identifiable information were anonymised before being imported into NVivo V. 12 Plus software,^20^ which was used to assist the organisation and coding of data. Reflexive thematic analysis was broadly interpretive and inductive.^17^ MP, TKM and KW met regularly to discuss interpretation and develop the coding framework. Outcome measures prioritised as being most important were given a score of 13, second most important a score of 12, third most important a score of 11 and so on down to a score of one. Outcomes were then ranked. Findings were considered against the Adapted Theoretical Framework of Acceptability (ATFA) for paediatric trials^11 21^ and Principles of Biomedical Ethics^22 23^ (in particular, autonomy, justice, beneficence and non-maleficence) and synthesised using a symbiotic empirical ethics approach^24^ to produce normative conclusions (eg, should a randomised controlled trial be conducted?)

## Findings

### Participant recruitment

**Figure 1:**
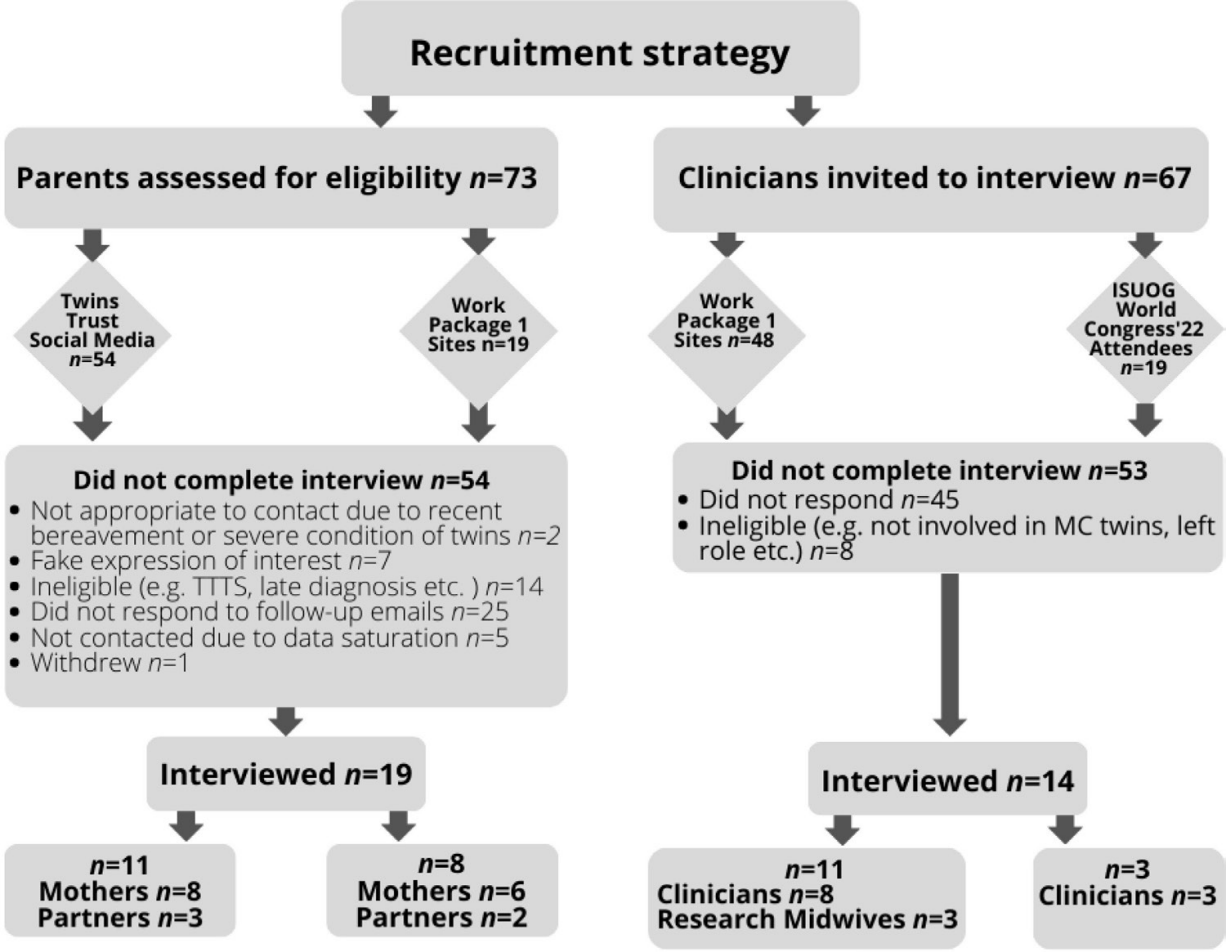
Participant recruitment. MC, monochorionic.

Seventy-three parents registered interest in taking part in an interview (figure 1). Recruitment was closed at the point of information power.^15^ Nineteen parents (14 mothers, 5 partners representing 28 babies and 14 families) took part in an online (n*=*11) or telephone (n*=*8) interview. Characteristics of the 19 parents and their pregnancy are shown in table 2.

**Table 2:** Parent characteristics

Parent characteristics (n*=*19)	
Hospital* where pregnancy was managed	Intervention sites (sites that perform selective termination or fetoscopic laser treatment for sFGR) (n*=*8)
	Local/referral sites (sites who do not perform selective termination or fetoscopic laser treatment for sFGR and refer to the above hospital sites) (n*=*5)
Gestation when sFGR diagnosed	16, 18, 20 and 22 weeks but noted (not diagnosed) in some as early as 12 weeks
Pregnancy management route taken	Expectant management (n*=*19)
Other management options offered	Selective termination (n*=*5, 3 families)Laser treatment (n*=*2, one initially for Twin-to-Twin Transfusion Syndrome)
Pregnancy outcome	Not known (n*=*8, 6 families-site parents, so pregnant at the time of the interview)
	Both twins lived (n*=*8, 6 families)Twin born with neurodevelopmental impairment (n*=*1)
	Both twins died (n*=*3, 2 families)
Country of residence	England (n*=*18)
	Scotland (n*=*1)
Ethnic group	Asian (n*=*2)
	Black Caribbean (n*=*1)
	Mixed Other (n*=*1)
	White British (n*=*15)

* Some mothers were cared for at multiple hospitals.

sFGRselective fetal growth restriction

Sixty-seven clinicians were invited to an interview. Recruitment was closed when 14 clinicians had taken part in an online (n*=*10) or telephone (n*=*4) interview (see table 3). Interviews with clinicians lasted between 53 and 83 min (mean*=*62 min), whilst parent interviews lasted between 47 and 106 min (mean 68 min).

**Table 3:** Clinician characteristics

Clinician characteristics (n*=*14)	
Roles	Involved in the clinical management of sFGR (n*=*11, including: Professors, Consultants or Specialists/Subspecialists in Obstetrics, Gynaecology, Multiple Pregnancy and/or Fetal Medicine)
	Research midwives (n*=*3)
Country where practising	England and Northern Ireland (n*=*11)
	The Netherlands (n*=*2)
	Germany (n*=*1)
Hospital sites	Intervention sites (n*=*7)
	Local/referral sites (n*=*7)
Involvement in the clinical management of sFGR	Between 2 and 6 years (n*=*4)
	Between 15 and 18 years (n*=*4)
	>20 years (n*=*5)
	N/A (e.g., research midwife, n*=*1)
Experience recruiting to trials	<2 years (n*=*2)
	Between 2 and 6 years (n*=*2)
	Between 10 and 15 years (n=4)
	>20 years (n*=*4)
	Not known (n*=*2)

NAnot applicablesFGRselective fetal growth restriction

Six interlinked themes will now be presented, which highlight the importance that parents and clinicians place on answering the research question and considering the practical and ethical challenges of conducting the proposed clinical trial.

### An important question to answer

Parents and clinicians indicated that the proposed trial would answer an important research question to guide clinical practice and discussions with parents:

I think it’s great that you’re doing something to help parents make decisions … there wasn’t really a lot of information … that me and my wife could find. (P14, partner, social media)

(Parents require) figures, so percentiles … because the science that is available at the moment is a bit contradicting and, in some aspects, also not always fitting to current practice. (C14, doctor)

Participants spoke of the need for evidence to alleviate the psychological distress that comes with making the ‘traumatic’ (P2, bereaved mother, social media), ‘impossible decisions’ (P1, bereaved partner, social media) about whether to go down the expectant management or intervention route, and for clinicians to confidently counsel parents about which route to take (C3, doctor). Clinicians stated that they ‘sometimes counsel too pessimistic’ and are ‘ashamed’ that they cannot provide parents with the right information specific to their pregnancy:

I sometimes am ashamed of that I have to say to parents, in this modern, developed world in which medicine can treat, let’s say, metastasis of melanomas with immune therapy, we cannot predict what the outcome is of their specific pregnancy complication. (C14, doctor)

### Pregnancy management decision-making as ‘traumatic’ when outcomes include death or serious disability of one or both twins

We began parent interviews exploring clinical practice and the pregnancy management route experienced. All parents had their pregnancy managed expectantly. Nine parents (six families) had considered selective termination and laser treatment options, yet described how their decision to decline intervention was informed by clinicians explaining how there were positive indicators of life (e.g., blood flow), which gave parents hope for the survival of both babies.

She (clinician) said, You can terminate little twin and focus on just having one baby, but because she had also said that all the internal blood flow was normal, that was an option which we said we weren’t going to take. (P12/13 joint interview, pregnant mother, site)

I don’t see why if we’ve got this far we can’t get further (…) I only need to make it to 28 weeks for them to be able to be born, even if it is very prematurely … I kind of felt like I didn’t want to do too much action. I felt protective of the pregnancy in a sense I didn’t want to do anything to upset it. (P11, mother, social media).

Although clinicians believed that parents should lead decision-making about whether to intervene, it was clearly evident in parent interviews (and compounded by whether their twins lived or died) that such decisions were traumatising, causing much distress and burden. They spoke of feeling disconnected from their surroundings, with bereaved parents stating ‘that some people end up being diagnosed with PTSD after having to make difficult decisions around their babies’ (P8, bereaved mother, social media). One mother who went on to have two healthy babies described her smaller twin as having ‘a life that wouldn’t need to be lost’ (P9, mother, social media).

Clinicians highlighted how the timing of intervention is difficult due to the changing and unpredictable nature of sFGR in MC twin pregnancy:

We can only offer selective reduction (termination) up to a certain gestation, usually 24 weeks. It’s a tricky one, because you think, ‘Well, in two weeks’ time, it is highly likely that the ductus venosus is going to become abnormal … By then, I may not have the option of offering them a selective reduction. And then, what if it dies?’ … And so, you can end up in this very difficult situation. If you don’t offer it early enough … but then you end up in this situation where you’re 24 to 25 weeks, you can’t offer a selective reduction, because technically, it’s not possible … I think that is the really difficult decision for them, because then it is an impact on their life or the life of that child. I mean, accepting death is so difficult, but looking after a child with a disability is a different ballgame altogether. So, it is those things that I think are very difficult for parents to weigh up. (C8, Doctor)

As well as the long-term impact of having conversations about selective termination, clinicians and parents were worried about the ‘devastating’ scenario where ‘it’s possible that one could’ve made a wrong decision … based on a worst-case scenario’ (P17, mother, site).

### The challenge of diagnosing an unpredictable condition in the context of a trial

Clinicians emphasised that every pregnancy is different and that there are many factors that determine their decision-making to recommend expectant management or active intervention. Clinicians’ decision-making was described as being ‘on a case-by-case basis’ (C12, doctor) and informed by multiple factors, including the severity and type of sFGR (as determined by Dopplers), as shown in Gratacós *et al* (2007) three types classification of sFGR^25 26^ (see table 4).

**Table 4:** Gratacós *et al* (2007) classification of sFGR

Pregnancy course	Description according to the Doppler finding of end-diastolic flow in the umbilical artery of the smaller twin	Potential outcomes
Type I	Persistently positive	At the lower end of the spectrum of severity. Type I has the best outcome in terms of mortality and morbidity (neurological damage) and is unlikely to require active intervention or early delivery
Type II	Persistently absent or reversed	Type II has worst prognosis. More severe, progressive deterioration that leads to considering active intervention or delivery in 90% of cases (earlier delivery may prevent mortality of one or both twins, but increases the risk of morbidity)
Type III	Intermittently absent or reversed	Better outcomes than type II cases, but still a highly unpredictable clinical course in terms of mortality and morbidity of both twins, requiring active intervention or early delivery in 10.8% of cases

sFGRselective fetal growth restriction

Other factors that determine clinicians’ decision-making to recommend expectant management, active intervention or early delivery were: the gestational age at diagnosis; the ‘size of the’ affected twin (C12, doctor); ‘the speed of growth decreases’ (C14, doctor) ‘where the placenta is’*/*placental vessels are (C14, doctor)/‘accessibility to the smaller twin’ (C7, doctor); and the perceived acceptability of intervention for parents. Furthermore, intervention in sFGR pregnancy ‘really varies according to … the centre where the patient is seen’ (C1, doctor), ‘the culture … (and) general consensus … of the population (area or country) and how they see things’ (C10, doctor), the knowledge and experience of, and how the individual clinician articulates the benefits and risks of pregnancy management options. These factors also contributed to clinicians’ suggestions for additions or amendments to the proposed inclusion and exclusion criteria (see online supplemental file 10).

While there was some variance in clinician’s preferred management approach, most clinicians stated they would not discuss intervention options early in the pregnancy, particularly for type I sFGR, and would ‘see again always … in one week’s time, just to see how things evolve’ (C2, doctor).

Parents described being told that their condition could correct itself or change from a more severe type to one with a more favourable outcome:

Like my consultant said, it’s a weekly thing. Today’s appointment is good, but we don’t know if it’s going to get worse or better next week. So, that was made very clear. (P17, pregnant mother, site)

When parents reflected on their discussions in these situations, they said they had relied on the clinician’s advice. However, this posed difficulties for parents who were faced with contradictory advice from different clinicians:

### Parents require clear information about risks and potential outcomes to make informed decisions as to whether to take part in the proposed RCT

When asked what would make an RCT like FERN more acceptable, parents said that they would want reassurance from their consultant that (hypothetically) taking part would ‘have no greater adverse outcome if we do this than if we didn’t do it?’ (P1, bereaved partner, social media) ‘because it’s such a … not invasive. Invasive is the wrong word. But it’s not, like, an observational study. It could actually affect what happened’ (P2, bereaved mother, social media).

Parents would require information and statistics about potential outcomes for each trial arm to decide whether or not to take part:

I don’t even know whether this exists, but potentially, statistics of how successful expectant management would be, how successful interventions are. So, the science behind each of the choices in simple numbers, so that it’s in black and white, easy to see how positive each of the outcomes are. (P4, mother (one twin with neurodevelopmental disability), social media).

Nevertheless, most parents and clinicians agreed that selective termination as a trial arm was not acceptable. While parents said that a trial comparing laser treatment with expectant management would be more acceptable because ‘you weren’t necessarily selectively choosing which baby would have to be terminated’ (P9, mother, social media), some clinicians said that they were ‘not a fan of laser because I think we have to be honest that we don't have any pathophysiological argument to say that it will improve the outcome’ (C2, doctor). Another clinician said that it would be unethical not to provide parents with the evidence about outcomes of laser treatment in sFGR:

It would be unethical … (to) not provide parents with the … current evidence (that) shows that they’re more likely to take a baby home if they have a selective reduction, compared to if they had a laser. (C8, doctor)

### The proposed RCT was viewed by parents and clinicians as ‘ethically murky’

While recognising the importance of answering the question about which management option is most effective for sFGR in MC twin pregnancy, our findings suggest that conducting an RCT comparing expectant management to active intervention would not be acceptable to parents, who view the proposed study as ‘ethically murky’ (P3, pregnant mother, site). Most parents clearly stated that they would not participate in an RCT with active intervention and expectant management as trial arms because they would not want the fate of their babies’ lives being left to a randomisation process:

If we were approached, I would be a straight 'No!' straightaway… Just reading the treatment sections (in the proposed participant information sheet), like the options of treatments, the termination treatments were definitely, immediately, I was like**,** 'Okay, no!' It was an immediate 'No!' That was it!. (P18, partner of pregnant mother, site)

Parents were clear that they would drop out of the proposed trial if they were randomised to a trial arm that they were not comfortable with, particularly if their pregnancy course was type I:

If it were us, I would’ve gone with it but if I was put into a category that I didn’t agree with I would’ve pulled out. Going back to the whole severity level of the pregnancy on a scale of one to ten, then being randomly put into category B which is termination, if you were on the less severe end of the spectrum you might look at that and think, 'I’m not happy with that!’. (P6, partner, social media).

Indeed, clinicians raised ethical concerns about randomising women to a trial arm, especially selective termination, that might not be appropriate to their individual case. Decision-making was informed by the severity and type of sFGR (see table 4):

It very much depends on the type of selective fetal growth restriction, whether it’s Type I or Type II … or Type III … We know that outcome for Type I is good without intervention. Outcome for Type II with abnormal ductus venosus is bad without intervention … Type II … deteriorates much faster and in a predictable way. And Type III can go on for a long, long time, but it’s an unpredictable, sudden loss. (C8, doctor)

### ‘Live birth’, ‘childhood disability’ and ‘neurodevelopmental impairment’ were the most important outcome measures for parents and clinicians

Parents and clinicians were asked to consider a list of 13 potential outcomes sent prior to interview and were asked if there were any additional outcomes that they felt were missing.

The ranking of outcomes prioritised by parents can be seen in table 5, and the ranking of outcomes prioritised by clinicians in table 6. Outcome measures prioritised as being most important were given a score of 13 and those ranked least important were given a score of one.

**Table 5:** Parent ranking of outcomes

Weighted ranking	Outcome	Weighted score	No. (& %) parents/18*
1	Live birth	234	18 (100)
2	Childhood disability†	96	9 (50)
3	Neurodevelopmental impairment†	89	8 (44)
4	Gestational age at birth	72	8 (44)
5	Child quality of life†	66	7 (39)
6	Birth weight	61	6 (33)
7	Loss during pregnancy or before final hospital discharge†	60	6 (33)
8	Death of surviving twin after death of co-twin†	54	5 (28)
9	Procedure-related adverse outcome	32	4 (22)
10	Intertwin birth-weight discordance	31	5 (28)
11	Parental stress†	30	7 (39)
12	Parent quality of life†	18	3 (17)
13	Length of stay in hospital	12	3 (17)

* aAs P19, pregnant mother, site is missing data.

† Most parents suggested these outcomes be grouped together and they ranked them togetherMost parents ranked the three outcomes of 'Childhood disability', 'Neurodevelopment impairment' and 'Child quality of life' equally and suggested that they be grouped together. Parents also ranked and suggested that the two outcomes of 'Loss during pregnancy or before final hospital discharge' and 'Death of surviving twin after death of co-twin' be grouped together. Additionally, the two outcomes of 'Parental stress' and 'Parent quality of life' were ranked together by parents, who suggested that they be grouped together.

**Table 6:** Clinician ranking of outcomes

Weighted ranking	Outcome	Weighted score	No. (& %) of clinicians/12*
1	Live birth (of at least one twin)	125	13 (93)
2	Neurodevelopmental impairment†	84	13 (93)
3	Childhood disability† (follow-up until at least 8 years old)	73	13 (93)
4	Death of surviving twin after death of co-twin†	67	10 (71)
5	Gestational age at birth (include short and long-term consequences of prematurity here or under new outcome ‘Neonatal morbidity’, which is currently missing)	66	11 (79)
6	Loss during pregnancy or before final hospital discharge+ (Define—does this mean one or two losses and does this include whether death is due to termination of pregnancy?)	66	10 (71)
7	Procedure-related adverse outcome+ (include premature rupture of membranes, pregnancy loss and injury to the fetus)	61	10 (71)
8	Birth weight (centile)	55	10 (71)
9	Intertwin birth-weight discordance	51	9 (64)
10	Parent quality of life†	50	7 (50)
11	Parental stress†	40	9 (64)
12	Length of stay in hospital†	36	9 (64)
13	Child quality of life†	36	7 (50)

* aAs C8 and C12 are missing data.

† *+ Need to be a composite/unable to separate this outcome from another/othersClinicians stated that the two outcomes of 'Neurodevelopment impairment' and 'Childhood disability' should be composite as they are unable to separate these outcomes from each other. Clinicians also suggested that the two outcomes of 'Death of surviving twin after death of co-twin' and 'Procedure-related adverse outcome' are unable to be separated and should be composite. Additionally, clinicians stated that the four outcomes of 'Parent quality of life', 'Parental stress', 'Length of stay in hospital', and 'Child quality of life' are composite and should be grouped together.

The most important outcome for the proposed trial for parents and clinicians was live birth which, for clinicians, meant the survival of one of the twins and for parents meant the survival of both twins, even if it meant that the twins had neurodevelopmental impairment:

I remember saying I would have preferred to have two alive children with … a bit of cognitive impairment … (or) disabilities than two dead ones or one dead one (P11, mother, social media).

Neurodevelopmental impairment and disability, which were the next most important outcome measures for parents and clinicians, had been presented to the participants as two separate measures. However, participants spoke of how these outcomes, together with the child’s quality of life (which was an important outcome measure to parents), overlap and could be measured together:

Parental stress was ranked as one of the least important outcomes (weighted 11 most important for parents and clinicians). Participants spoke of how stressful going through a high-risk pregnancy was for families, and as stress is ‘almost like a given’ (P15, pregnant mother), they would not consider this an important outcome to be measured in the proposed RCT.

Almost half of clinicians, unprompted, said that ‘neonatal morbidity’ meaning ‘all of the complications that can arise in the neonate, while the neonate did not actually die’ (C6, doctor) was a missing outcome, with one clinician saying that that neonatal morbidity is an outcome that is included in other UK sFGR and neonatal trials.

In other UK trials of sFGR and neonatal trials, there is actually quite a long list of neonatal morbidity outcomes or indicators that should be included in this list. (C1, doctor).

Suggestions for longer term (and missing) outcome measures were made by both clinicians and parents, such as, importantly, including ‘some sort of measurement of parental experience or any regret or anything to do with their decision-making’ (C3, doctor) for trial participation, parent emotional well-being and living with the choices made (e.g., post-traumatic stress disorder and suicide risk), as demonstrated by this mother’s powerful question:

Did they (parents) survive emotionally … after the decisions … to terminate one of these kids? … (Did) parents … go and kill themselves because they have made the wrong decision? How the hell do you, as a mother, cope? (P16, pregnant mother, site)

Clinicians spoke of how ‘parents will always remember that they were offered a termination’ (C8, doctor) and ‘are (still) traumatised by mentioning the option of cord occlusion’ when their child is followed up at age 8 years:

It is so difficult, and that still, at the age of eight years, they (parents) look at the twins and they think frequently about one of them that they had, that they could end up in a situation that they had chosen cord occlusion …. (C14, doctor)

### The proposed RCT is ‘like mission impossible’ and an alternative study design is required

After considering the proposed trial, participants stated that the FERN study would not be acceptable nor practical to conduct ‘in a randomised fashion’ (C2, doctor) as the risk of distress and burden for parents and harm or death to one or both twins would be too great. Applying our findings to the Principles of Biomedical Ethics^22 23^ (see online supplemental file 11) and ATFA for paediatric trials^11 21^ (see online supplemental file 12) and synthesising them using a symbiotic empirical ethics approach^24^ clearly support our findings that the proposed RCT would not be ethical or acceptable for clinicians or parents. As one parent said: ‘You can’t be ethical basically, I don’t think … It is almost like the mission impossible and you just need to find a way to kind of … There will be damage basically, you can’t avoid it, there is no way, there is no other way’ (P18, partner, site).

Some parents and clinicians suggested consideration should be given to other study designs that are more acceptable and still scientifically valid. One parent asked, ‘Is there another way of doing it? … Perhaps, for example, could those who are already going to have these managements, then base it on that, instead of selecting at random?’ (P5, mother, social media). Cohort studies were proposed by clinicians as an option ‘where patients are counselled in a similar way and then depending on what’s technically possible and also on patient preferences, you document an outcome in a uniform way. I think that’s the only way to do that’ (C2, doctor).

## Discussion

To our knowledge, this qualitative study is the first to explore parents and clinicians’ views on the acceptability and feasibility of conducting an RCT of active intervention versus expectant management in MC twin pregnancy with early-onset sFGR. To navigate the ethical issues with the proposed RCT, we drew on the ethical principles of autonomy, justice, beneficence and non-maleficence proposed by Beauchamp and Childress^22^ and involved an ethicist (RA) in the analysis of findings.

Our findings suggest that an RCT comparing active intervention versus expectant management would not be acceptable, seen as ethical to parents and clinicians, nor feasible to conduct. One of the main challenges to conducting the proposed RCT related to the different types, severity and clinical uncertainty around the diagnosis and management of sFGR in MC twin pregnancies. A recent retrospective study that assessed the accuracy of diagnosis of sFGR with Doppler ultrasound in MC twin pregnancies between 14 and 26 weeks of gestational age, in 280 pregnant women (118 with sFGR), found that second trimester Doppler and ultrasound measurements could correctly identify 74.5% of sFGR twins.^27^ However, the study did not report on the three types of sFGR, which correspond with different clinical behaviour, patterns and outcomes^25^ (see table 4). Type I and III sFGR have a better outcome than type II (albeit that type III has a highly unpredictable clinical course in terms of mortality and morbidity of both twins, requiring active intervention or early delivery in 10.8% of cases).^25 26^ Consequently, while all parents and clinicians spoke about the need for high-quality information to inform decision-making and were supportive of a study that aims to answer the FERN research question, many strongly opposed having selective termination as a trial arm, feeling that it would be unethical to randomise women with type I and type III sFGR to a trial arm ‘directly killing’^28^ their sFGR twin.

Similarly, although parents in our study considered laser treatment to be more acceptable as a trial arm than selective termination, because they believed that this pregnancy management option would minimise the risk of harm to the sFGR twin (and they would not be ‘directly killing’^28^ their sFGR twin), this was in contradiction with clinicians’ views on laser treatment as a trial arm. Whilst laser treatment is a common and effective option for Twin-to-Twin Transfusion Syndrome,^29^ the evidence to support it as an effective therapy for sFGR is currently lacking. Clinicians felt that it would be unethical to not provide parents with the evidence that shows that they are more likely to take a baby home if they have a selective termination, compared to if they have laser treatment.

Furthermore, our findings suggest it would be unethical, and in conflict with the Hippocratic Oath promise of ‘first, do no harm’ and the fundamental ethical principles of non-maleficence and beneficence,^30^ to randomise women with type I and type III sFGR, who would potentially not require intervention, to receiving laser treatment intervention. A mother with type II sFGR that may require active intervention could potentially be randomised to the expectant management arm of the proposed RCT. The ethical principles of non-maleficence and beneficence demand that patients be offered care that minimises risks. However, in the proposed FERN RCT, acting for the rights of one twin diminishes the rights of the other twin, which is in opposition to the ethical principle of individual autonomy for both twins^31^ and, depending on opinion about when a fetus becomes a child with a right to life, survival and development, the United Nations Convention on the Rights of the Child.^32^ This poses issues as, for the women in our study who ultimately gave birth to two healthy babies, terminating the life of their sFGR twin/both twins would have been, as one mother said, ‘a life that wouldn’t need to be lost’. Therefore, clinicians must be clear about the benefits and risks of each pregnancy management option when counselling parents, especially as parents ranked survival of both twins as the most important outcome. Our findings also highlight the importance of the long-term measurement of parent well-being and any regret to do with their decision-making, even though long-term parent related outcomes of trial participation are rarely collected.^33^

We also found that clinicians’ practice varied regarding active intervention and the timing of discussion with parents about this (depending on the culture of the population in the area that they are practising in). This has implications for the proposed RCT, as some parents would not typically need to discuss active intervention. Although clinicians believed that parents should lead decision-making about pregnancy management in line with the biomedical ethical principle of autonomy,^22 34 35^ it was clearly evident that even the offering of selective termination and suggestion that parents should make a decision, which was traumatic for parents and potentially caused long-term harm, is in breach of the non-maleficence biomedical ethical principle, regardless of outcome or pregnancy management route taken. One parent spoke of having feelings of dissociation during the conversation, which puts into question whether parents will truly have the capacity to make an informed consent decision about the proposed RCT. This finding demonstrates the difficulties in balancing the biomedical ethical principles of autonomy, beneficence and non-maleficence, as well as the need to answer an important research question to inform future clinical practice to improve outcomes for such challenging pregnancies. Shea argues the need for specification and balancing to determine the relative weight of conflicting principles.^34^ Ultimately, the psychological long-term impact for parents of having to make a decision that may result in the death or severe damage to one or both twins must be considered, and parents must be counselled in a way that helps them to manage feelings of guilt, grief and mental health distress. This recommendation is relevant to future clinical practice as well as studies they may be conducted in the future.

### Conclusion

Our findings have shown that parents and clinicians do not consider an RCT comparing active intervention versus expectant management to be acceptable or ethical for the management of MC twin pregnancies complicated by sFGR. Drawing on findings from the wider FERN study, as well as the barriers identified in this study to both recruitment and retention, alternative study designs such as an international multicentre observational cohort study or propensity score matching should be considered to address this important research question. Parents value clear information about potential risks and outcomes to make better informed decisions and clinicians wish to be in a better position to counsel parents appropriately. As we have shown, care should be taken when counselling parents as the impact of such clinical discussions can have long-lasting effects on parents, regardless of outcome.

### Strengths and limitations of this study

This study provides insight into how parents and clinicians would respond to being invited to participate in an RCT investigating active intervention versus expectant management of MC twin pregnancies complicated by sFGR. The primary strength of the study is the recruitment of parents whose experiences varied in terms of pregnancy outcomes. The sample included bereaved families who had lost both twins, those who had two healthy twins, a case where the co-twin had neurodevelopmental impairment and women who were currently experiencing an MC pregnancy complicated by sFGR and their partners. This last group, in particular, provided insight into how women and their partners might respond to being asked to participate in a definitive trial. Another strength of this study was the involvement of an ethicist in the analysis of findings. Several factors that would help with the design of a future study that does not include randomisation were identified. Our findings can help clinicians reflect on how best to carry out pregnancy management conversations with women to ensure that they are aware of the risks and benefits of each option.

One of the study limitations was that only parents who had their pregnancy managed expectantly took part in an interview (although six families had been offered active intervention). Thus, we cannot conclude that our results accurately reflect the views of women who experienced selective termination or laser treatment intervention. The sample consisted primarily of families of White British ethnicity and all interviews were conducted with English-speaking participants. The views, experiences and understanding of the decision-making parents are asked to make should be explored with families via an interpreter. Although research samples should always reflect the diversity in the population studied, this is particularly important in this study where parents will have to make decisions that include the termination of one of their twins. As demonstrated by our findings, pro-life or pro-choice views on selective termination can be influenced by cultural and religious backgrounds.^36 37^ Interviews were conducted over the telephone or online which may have impacted on possible eye contact or development of rapport. However, our previous research and other studies have reported that telephone interviews are preferred over face-to face interviews when discussing delicate topics or balancing childcare responsibilities.^38^

## supplementary material

10.1136/bmjopen-2023-080488 online supplemental file 1

10.1136/bmjopen-2023-080488 online supplemental file 2

10.1136/bmjopen-2023-080488 online supplemental file 3

10.1136/bmjopen-2023-080488 online supplemental file 4

10.1136/bmjopen-2023-080488 online supplemental file 5

10.1136/bmjopen-2023-080488 online supplemental file 6

10.1136/bmjopen-2023-080488 online supplemental file 7

10.1136/bmjopen-2023-080488 online supplemental file 8

10.1136/bmjopen-2023-080488 online supplemental file 9

10.1136/bmjopen-2023-080488 online supplemental file 10

10.1136/bmjopen-2023-080488 online supplemental file 11

10.1136/bmjopen-2023-080488 online supplemental file 12

## Data Availability

No data are available.
