# Supplementary material for: Balancing key stakeholder priorities and ethical principles to design a trial comparing intervention or expectant management for early-onset selective fetal growth restriction in monochorionic twin pregnancy: FERN qualitative study
Source: BMJ Open. 2024 Aug 9;14(8):e080488. doi: 10.1136/bmjopen-2023-080488 (PMC11331883; doi:10.1136/bmjopen-2023-080488)
Supplement: online supplemental file 2 [file bmjopen-14-8-s002.pdf]

Participant Information Sheet

FERN: Intervention or Expectant Management for Early Onset Selective Fetal Growth Restriction in Monochorionic Twin Pregnancy

Birth partner interviews

We are inviting the birth partners of women pregnant with twins to take part in a research study. Before you decide whether or not to take part it is important for you to understand why the research is being performed and what it will involve. Please take the time to read the following information carefully and discuss it with others if you wish. A member of our research team will go through the information sheet with you and answer any questions you may have. Please take time to decide whether or not you wish to take part.

Thank you for reading this.

Why are we doing the study?

The UK has approximately 11,000 twin pregnancies per year with a third of these pregnancies sharing a placenta (monochorionic (MC) twins). MC twin pregnancy presents extra risks to both the mother and the babies, with some babies dying during pregnancy or shortly after birth. Often this is due to a complication called selective Fetal Growth Restriction (sFGR), where one twin is smaller than the other. sFGR affects one in seven MC twin pregnancies in the UK although we know less about pregnancies where this happens early (before 24 weeks).

There are three main ways of managing MC twin pregnancies with sFGR: 1) a watch and wait approach (also called expectant management), 2) a procedure that blocks the umbilical cord from the smaller twin to the placenta and causes the loss of the smaller twin (also known as selective termination), and 3) a laser that can be used to completely separate the twins' circulations. All of which present significant risks (death and severe disability) to one or both twins.

At present there is a lack of evidence to tell us the best way of managing sFGR in MC twin pregnancies. Currently, women and their partners are offered different management options depending on where they live and who they see. It is also clear that there are gaps in what we know about sFGR.

To be able to find the best way to manage these pregnancies there is much need for a clinical trial comparing management options. Before running a trial, we need to understand things like how many twin pregnancies would be needed to run the trial and whether parents would think such a trial is acceptable. We also need to work out which management options would be the best to use and what outcomes would be important.

To try to do this we would like to hear about your and your partners pregnancy experience and find out your views and opinions on a potential future clinical trial by inviting you to take part in an interview.

Participant Information Sheet

Why have I been chosen?

We are inviting birth partners of women aged 18 years and older who are currently pregnant (16 - 23 weeks) with an MC twin pregnancy complicated by sFGR or have had an sFGR affected MC twin pregnancy in the last 3 years to take part in this study.

Do I have to take part?

It is up to you to decide whether or not to take part in this study. If you decide to take part - you will be asked to sign a consent form. You will be free to withdraw from the study at any time, without giving a reason. If you decide not to take part – this will not affect the care you or your family receives in any way.

What will happen to me if I take part?

If you agree to take part in the study, you will be asked to provide your contact details so that a member of the research team can get in touch with you to arrange a date for your interview. If you have any questions about the study, please contact Dr. Kerry Woolfall (Tel: 0151 794 4634, Email: k.woolfall@liverpool.ac.uk). Your interview can be carried out over the telephone, online (via Microsoft Teams or Zoom) or face to face (in line with the latest government guidance on COVID-19), whichever you prefer. It will last approximately 40 minutes and will be arranged at a time that is suitable for you. Before your interview starts you will be asked to give your permission for your conversation to be recorded. This is so that we have a record of your consent if you are having a telephone or online interview. It will also allow us to analyse the information you provide to us at a later date. Due to your experience during pregnancy you may find some parts of the interview upsetting. You are free to decline to answer any questions you do not wish to or to stop the interview at any point. Your interview will be carried out by experienced researchers and any distress will be treated with care and compassion.

How will you use my data?

How will you use information about me?
We (study sponsor – the University of Liverpool) will need to use information from you for this research project.

This information will include your initials, name, contact details (telephone number and email address), and the first part of your postcode. People will use this information to do the research or to check your records to make sure that the research is being done properly.

People who do not need to know who you are will not be able to see your name or contact details. Your data will have a code number instead.

We will keep all information about you safe and secure.

Once we have finished the study, we will keep some of the data so we can check the results. We will write our reports in a way that no-one can work out that you took part in the study.

Participant Information Sheet

What are my choices about how my information is used?

- You can stop being part of the study at any time, without giving a reason, but we will keep information about you that we already have.
- We need to manage your records in specific ways for the research to be reliable. This means that we won't be able to let you see or change the data we hold about you.

Where can I find out more about how my information is used?
You can find out more about how we use your information

- at www.hra.nhs.uk/information-about-patients/
- our leaflet available from www.hra.nhs.uk/patientdataandresearch
- by asking one of the research team
- by sending an email to legalservices@liverpool.ac.uk, or
- by ringing us on 0151 795 0523.

What are the possible benefits of taking part?

Taking part in this study will have no direct benefit to you or your partner. It will however, in the long-term, result in a better understanding of the outcomes for sFGR in MC twin pregnancies managed in a variety of ways. This will not only benefit women in terms of counselling as to which pregnancy management option to choose, but will also provide the much needed evidence to design and conduct a future clinical trial comparing these management options. The ultimate goal of such a trial is to establish the best possible way to manage MC twin pregnancies complicated by sFGR.

What are the possible risks of taking part?

Taking part in this study presents no direct risks to you and your partner. All the information you provide to us will be collected, stored and used in compliance with data protection regulations (GDPR) and the study will be conducted in accordance with ethical and legal practices.

What will happen if I don't want to continue in the study?

You are free to withdraw from the study at any time, without explanation. The care you or your family receives will not be affected in any way. If you withdraw from the study we will not collect any further information from you. We will however keep and use any information you have already provided.

What will happen to the results of the research study?

It is intended that once the study is complete the results will be used to establish whether a future clinical trial comparing different management options for sFGR in MC twin pregnancies is both feasible and acceptable. We will also use the information you provide to us to help with the design of the trial.

Participant Information Sheet

Where can I get further information or discuss any problems?

If you have any questions or concerns about any aspect of this study, please contact a member of the research team (Email Mariana on M.Popa2@liverpool.ac.uk; Tracy on Tracy.Mitchell@liverpool.ac.uk; or Kerry on k.woolfall@liverpool.ac.uk). If your concerns are not resolved, you can visit the Patient Advisory Liaison Services (PALS) by asking at your hospital reception. If you should need additional support to help with any distress arising from your pregnancy you can contact either the Twins Trust (email: support-team@twinstrust.org or telephone: 01252 332 344) or your GP.

Who is organising and funding the research?

The National Institute for Health Research (NIHR) Health Technology Assessment (HTA) programme is funding this study (Reference: HTA-128596) and Professor Asma Khalil is the study Chief Investigator. The study is sponsored by The University of Liverpool and is managed by the Harris Wellbeing of Women Research Centre, University of Liverpool.

Who has reviewed the study?

All research in the NHS is looked at by an independent group of people, called a Research Ethics Committee, to protect your interests. This study has been reviewed for ethical considerations and given a favourable opinion by members of the South West – Cornwall and Plymouth Research Ethics Committee.

Contact for further information.

Should you have any further queries regarding this study, please contact:

Professor Asma Khalil, Chief Investigator, University of Liverpool / Liverpool Women's NHS Foundation Trust, Crown Street, Liverpool, L8 7SS. Email: fern1@liverpool.ac.uk Tel: 0151 795 9565.
