# Supplementary material for: Balancing key stakeholder priorities and ethical principles to design a trial comparing intervention or expectant management for early-onset selective fetal growth restriction in monochorionic twin pregnancy: FERN qualitative study
Source: BMJ Open. 2024 Aug 9;14(8):e080488. doi: 10.1136/bmjopen-2023-080488 (PMC11331883; doi:10.1136/bmjopen-2023-080488)
Supplement: online supplemental file 3 [file bmjopen-14-8-s003.pdf]

Participant Information Sheet

FERN: Intervention or Expectant Management for Early Onset Selective Fetal Growth Restriction in Monochorionic Twin Pregnancy

Practitioner interviews and focus group

We are inviting you to take part in a research study. Please take time to read the following information and ask us if there is anything that is unclear or if you would like further details about the study (contact details overleaf).

Why are we doing the study?

At present, there is a lack of evidence to inform the best way of managing Selective Fetal Growth Restriction (sFGR) in monochorionic (MC) twin pregnancies, particularly where this happens early in pregnancy (before 24 weeks). Currently, women and their partners are offered different management options depending on their geographical location and their clinical team. It is also clear that there are gaps in our knowledge in terms of sFGR.

There is a need for a clinical trial comparing different management options for sFGR. Before conducting a trial however, several factors such as the number of twin pregnancies needed to power the trial and whether women and clinicians think such a trial is acceptable and would be willing to participate need to be determined. We also need to establish the best management options to use and the most important outcomes.

We are conducting the FERN feasibility study with the aim of addressing these questions in order to establish the feasibility and design of a clinical trial. We would like to invite you to take part in an interview with one of our team. We may also conduct focus groups with MC twin parents and clinicians if interviews show differences in opinion about the study.

Why have I been chosen?

As you are involved in the care of sFGR in MC twins your views on a potential future clinical trial are important.

What will happen if I take part?

We will ask you to register your interest in taking part in either an interview or a focus group. The email invitation outlines which type of interview we are recruiting to at this point in time. Interviews will take place by telephone, online (via Microsoft Teams or Zoom) or face to face for participants in the North West (in line with the latest government guidance on COVID-19) and will be arranged at your convenience. Interviews will take about 40 minutes. Focus groups will take place online or in a private meeting room at one of the participating FERN research sites (in line with the latest government guidance on COVID-19). The focus group will take about 60 minutes and involve 8-10 site healthcare professionals. All interviews and focus groups will be conducted by the University of Liverpool FERN study team.

Participant Information Sheet

What will I be asked about?

Interviews and focus groups will explore your views on:

- trial design, including views on active intervention and expectant management, randomisation, outcomes, and approach to recruitment and consent, including consent decision making and length and content of trial information materials,
- factors influencing parent and clinician decision-making when potential outcomes include death or serious disability of one or both twins, and
- acceptability of a future trial, including potential barriers to recruitment, consent decisions, trial procedures, equipoise; inclusion / exclusion criteria and training needs.

How will you use my data?

How will you use information about me?
We (study sponsor – the University of Liverpool) will need to use information from you for this research project.

This information will include your initials, name, and contact details (telephone number and email address). People will use this information to do the research or to check your records to make sure that the research is being done properly.

People who do not need to know who you are will not be able to see your name or contact details. Your data will have a code number instead.

We will keep all information about you safe and secure.

Once we have finished the study, we will keep some of the data so we can check the results. We will write our reports in a way that no-one can work out that you took part in the study.

What are my choices about how my information is used?

- You can stop being part of the study at any time, without giving a reason, but we will keep information about you that we already have.
- We need to manage your records in specific ways for the research to be reliable. This means that we won't be able to let you see or change the data we hold about you.

Where can I find out more about how my information is used?
You can find out more about how we use your information

- at www.hra.nhs.uk/information-about-patients/
- our leaflet available from www.liverpool.ac.uk/legal/data_protection/
- by sending an email to legalservices@liverpool.ac.uk, or
- by ringing us on 0151 795 0523.

Participant Information Sheet

What are the possible benefits and risks of taking part?

This qualitative component of the FERN study is very low risk. Should you want to discuss any aspect of the study, please contact Kerry Woolfall (details below). Findings of this study will be used to inform the design of a future clinical trial. We cannot promise that you or the families you work with will benefit directly from this study, but many people find that taking part in studies of this sort is useful because they have a chance to reflect and air their views.

Who is involved in this study?

The National Institute for Health Research (NIHR) Health Technology Assessment (HTA) programme is funding the study (Reference: HTA-128596) and Professor Asma Khalil is the FERN study Chief Investigator. The study is sponsored by The University of Liverpool and managed by the Harris Wellbeing of Women Research Centre, University of Liverpool. Dr Kerry Woolfall (University of Liverpool) is leading this qualitative component.

What if there is a problem?

Any complaint about the conduct of this study, the way you have been dealt with during the study or any possible harm you might suffer will be addressed. If you have a concern about any aspect of this study, then please speak to a member of the FERN study team who will do their best to answer your questions (see contact details below). If you remain unhappy and wish to complain formally, then you can do this through the NHS Complaints Procedure. Details can be obtained from your employer.

Contact for further information.

If you have any further questions about the interviews or focus groups, please contact:

Dr. Kerry Woolfall, Qualitative Lead, University of Liverpool, Block B Waterhouse Building, 3 Brownlow Street, Liverpool, L69 3GL. Email: k.woolfall@liverpool.ac.uk Tel: 0151 794 4634.

Or

Professor Asma Khalil, Chief Investigator, University of Liverpool / Liverpool Women's NHS Foundation Trust, Crown Street, Liverpool, L8 7SS. Email: fern1@liverpool.ac.uk Tel: 0151 795 9565.
