# Supplementary material for: Balancing key stakeholder priorities and ethical principles to design a trial comparing intervention or expectant management for early-onset selective fetal growth restriction in monochorionic twin pregnancy: FERN qualitative study
Source: BMJ Open. 2024 Aug 9;14(8):e080488. doi: 10.1136/bmjopen-2023-080488 (PMC11331883; doi:10.1136/bmjopen-2023-080488)
Supplement: online supplemental file 4 [file bmjopen-14-8-s004.pdf]

Topic guide for interviewing mothers and birth partners (SOCIAL MEDIA)

**Introduction, obtaining consent and any questions**
**Demographics**
- For administration reasons please could you tell me your:<br>DOB *(check that they are over 18 years)*,<br>Occupation,<br>First part of post code,<br>Ethnic background,<br>How many children do you have and their ages?<br>Where you saw the study advertised?
**Monochorionic (MC) Pregnancy Experience**
If known: My notes from when you registered interest in taking part in this interview state that you had identical twins in (*insert month and year*). Is that correct?<br>• Please tell me a little bit about your pregnancy?<br>• When were you admitted to hospital? *(Prompt confirm gestation in weeks)*<br>• At what point where you made aware that there may be a problem with your pregnancy and that one of your twins was smaller than the other? *(explore how and when this was explained).*<br>• Who spoke to you about the problem with your pregnancy?<br>• Can you recall what they told you?<br>• How did the doctor explain the options you had at that point in time? *(Explore: What the options were and at what timepoint these were presented? (e.g. Wait and see, selective termination [cord occlusion] or laser treatment- note only one option may have been presented so amend prompts below accordingly).*<br>- How were you feeling at that point in time?<br>- What information was presented to you at that point in time to help to inform your decision? *(e.g. written information or numbers/probabilities of survival)*<br>- Where any potential risks discussed for the options presented? *(if so, what risks were discussed for the options presented?)*<br>- Where any potential benefits discussed for the options presented? *(if so, what advantages or benefits were discussed for the options presented?)*<br>- Did you discuss potential options with anyone else?<br>- What type of things did you consider when making the decision about which option to take?<br>• Could you tell me what happened next? *(Leave this to parent to discuss and tell as much as they would like. Ultimately, we need survival information (may already have this from background question) and how child/children are now).*
**FERN Study (refer to draft information sheet)**
Have you had chance to look at the draft participant information sheet I sent to you for the proposed FERN study? (If no- read through sheet with parent)<br>Based on the participant information sheet please describe your understanding of what the FERN study is aiming to do?<br>What are your initial thoughts about this proposed study?<br>Would you have any concerns about the FERN study? *(Prompt: after exploring concerns- would you raise these concerns with the nurse or doctor?)*<br>Would you have any questions about the FERN study?<br>Looking at the information sheet, are there any parts of the study design that you think parents may find difficult to understand? *(explore language/jargon).*

Are there sections of the information sheet which you would prioritise when making your decision about whether or not to consent? *(Prompt: were there any parts of the information sheet that stood out to you in terms of influencing your decision as to whether or not you would like to take part?)*<br>Is there anything you would find useful when deciding whether or not you would like to take part?)<br>What kind of support do you think you would need when deciding whether to participate or not in the trial?<br>Who else would you need to talk to before making your decision? *(doctors, midwives, research nurses, partner, other family members, friends?)*<br>Would there be any other information that you would need to help make this decision?<br>Would you have given your permission to take part in the FERN study?... Could you tell me a bit more about your reasons for this?<br>How would you feel if you were randomised to 'watch and wait' (expectant management) in the proposed FERN trial?<br>How would you feel if you were randomised to receive cord occlusion (selective termination) in the proposed FERN trial?<br>How would you feel if you were randomised to receive laser treatment in the proposed FERN trial?<br>*[If cord occlusion doesn't seem like a viable option],* How would you feel if the study were to only have two options – 'watch and wait' and laser treatment? Would this make you more likely to hypothetically take part in the trial?<br>When do you think is the best time to approach a family to discuss the FERN Study? *(Prompt: we are suggesting within 24 hours)*<br>How much time would you need to consider the information before making a decision about the FERN Study?<br>Who do you think would be the best person to approach a family and when should this person go back or call the family again? *(look into where potential participants would be).*
**Outcomes**
As we have discussed, in the FERN study we want to find out the best way to manage monochorionic (MC) twin pregnancies.<br>To do this we will collect information *on (read through outcome measures list sent prior to interview).* By collecting information on these main things, we hope to find out which approach to managing MC twin pregnancies should be used in the future. These are called outcome measures.<br>It is important that we include outcome measures that matter to parents.<br>Thinking about your pregnancy what would you hope the outcome of your pregnancy management would be? (*Prompt: what effect would the approach to managing your pregnancy have to be most effective?*<br>- What would you be looking for as an indicator that your pregnancy was being managed well?<br>- What do you think about the outcome measures (*re-cap measures in the list provided*)?<br>- Is there another outcome measure that you think is important to families which we should be collecting information about in the FERN Study? (*prompt: present identified outcome measures based on responses to first two questions*)<br>Recap on outcomes measured and ask them to put in order of importance *(e.g. So far, you have mentioned x outcomes, X, Y & Z. Which would you say is the most important for this study? Second most important for this study?)*

Finally, is there anything else you would like to say about this proposed new study?
