# Supplementary material for: Balancing key stakeholder priorities and ethical principles to design a trial comparing intervention or expectant management for early-onset selective fetal growth restriction in monochorionic twin pregnancy: FERN qualitative study
Source: BMJ Open. 2024 Aug 9;14(8):e080488. doi: 10.1136/bmjopen-2023-080488 (PMC11331883; doi:10.1136/bmjopen-2023-080488)
Supplement: online supplemental file 5 [file bmjopen-14-8-s005.pdf]

INTERVIEW TOPIC GUIDE FOR PRACTITIONERS

FERN: Intervention or Expectant Management for Early Onset Selective Fetal Growth Restriction in Monochorionic Twin Pregnancy

Introductions and obtaining consent for audio recording

Section 1. Role and Background

1.1 Please tell me what your role is
1.2 How long have you been involved in the clinical management of sFGR?
1.3 How much, if any, experience do you have in recruiting to clinical trials?

Section 2: Current Practice

2.1 Could you please describe to me what your usual practice is for the management of sFGR in MC twin pregnancies with early-onset (prior to 24 weeks) sFGR?

Prompt: explore responses in reference to the management options in the box above. Explore: which options are preferred or most commonly used by you / your unit. If a particular option isn't used explore why (e.g. personal preference, not current practice on unit, or any barriers such as resources to provide a particular option)

2.2 What factors do you consider when making decisions about which management option would be most appropriate for individual patients?

Explore: what influences your decision making when death or serious disability of one or both twins are potential outcomes?

2.3 Who do you consult with when making these decisions?

Explore: which clinical colleagues

2.4 At what point/when do you think is the best time to approach mothers/partners to discuss management options with them?

2.5 I'm sure these are very difficult conversations to have with mothers / partners. Do you or your colleagues present a number of options for them to consider?

Explore: which management options do you present them with? Explore, which and what information is provided in these discussions

2.6 How do you present the benefits and risks of each option presented/what wording do you use? *(identify the wording used to present the risks and benefits of the three (if applicable) options)*

2.7 Could you tell me if there is anything that mothers and partners tend to prioritise when making a decision about pregnancy management options?

2.8 What questions do they ask?

2.9 Do they need any support or additional information when making this decision?

Section 3: Role and Involvement in the FERN Feasibility Study

3.1 Have you been involved in the WP1 – collecting prospective data on the management and clinical outcomes of MC pregnancies complicated by sFGR? ***(If no go to Section 3.6 - questions about inclusion and exclusion criteria)***

3.2 Which elements of this work package have you been involved in? Explore: screening patients, consenting patients

If screening:

3.3 Could you talk me through the screening process?
3.4 Have any potential participants been missed? Please elaborate
3.5 Do you think the screening process could be improved if we moved to a full trial comparing intervention versus expectant management of sFGR in MC twin pregnancy? (Yes/No) *Discuss potential challenges to screening or systems that have been put in place to assist screening.*

Inclusion and Exclusion Criteria

3.6 Do you have any comments or suggested changes for proposed inclusion and exclusion criteria that we sent you by email? Do you have this to hand?

3.7 Are there patients you think we should definitely **NOT** include in this proposed trial? *Explore who, why or why not*

Section 4: Defining Management Options (section to be informed and developed in light of any key WP1 findings of relevance)

I have a few questions about your unit's clinical management for these pregnancies

4.1 How often do you repeat the ultrasound in these pregnancies (e.g. once or twice/week)?
4.2 What is the gestation at delivery?
4.3 What are the triggers for delivery or intervention?
4.4 If you do need to intervene, which form of intervention do you use, selective termination or laser treatment? (if not clear, ask)
4.5 What determines your choice of a particular intervention?

CONSULTANTS ONLY

4.6 What is your management protocol if complications occur:
a) single intrauterine death (IUD) if the pregnancy is managed expectantly,
b) Single IUD if the pregnancy was treated by laser,
c) Preterm Premature Rupture Of Membranes (PPROM) if the pregnancy was treated by laser or cord occlusion,
d) the pregnancy developed twin anaemia polycynthemia sequence (TAPS),
e) the pregnancy developed TAPS after laser or cord occlusion.

4.7 Does your personal clinical management of such pregnancies differ from your unit's management protocol?
Explore: how and examples of what informs decision making

Section 5. Acceptability and Trial design

Check if the participant is familiar with the proposed trial design:
Women aged 18 years and older who are pregnant (16-23 weeks) with an MC twin pregnancy complicated by sFGR will be invited to participate in the FERN study. If they agree to take part in the study we will use a computer to decide at random which management option 1), 2) or 3) is followed.
1) Expectant: close monitoring but no active intervention. This carries a risk of death of the smaller twin. Death of the smaller twin may result in demise of the larger twin (40%) or disability (30%).
2) Selective termination of the smaller twin. This may protect the larger baby from harm if the smaller twin were to subsequently die. However, termination may not be acceptable to some parents.
3) Selective placental laser photocoagulation of connecting vessels. This is likely to be a complex surgery where Laser is used to close the connections between the babies in the placenta. It may worsen outcomes for the smaller twin.

5.1 Given the proposed trial arms, how acceptable would you find expectant management (e.g. close monitoring but no active intervention) of the sFGR twin as a trial arm?
Explore reasons including anything that would make this arm of the trial more or less acceptable

5.2 At the moment, the trial is exploring selective termination and laser treatment as two separate intervention arms. How acceptable would you find selective termination (e.g. cord occlusion) of the sFGR twin as a trial arm?
Explore reasons including anything that would make this arm of the trial more or less acceptable.

5.3 How acceptable would you find selective laser treatment as a trial arm? *Explore reasons including anything that would make this arm of the trial more or less acceptable*

5.4 How acceptable do you think it is that the decision about which treatment option in the intervention arm is made by a consultant or the parent?

5.5 Would you be willing to randomise women to the proposed FERN RCT?
Explore: any concerns about randomising patients to either expectant management or active intervention being different from how they would personally manage such pregnancies.
The following is informed by the discussion around the acceptability of the trial design, especially If proposed trial/trial arms do not seem acceptable:

5.6 Is there an alternative trial design that you'd suggest?

Section 6. Parents discussion

6.1 Considering the suggested trial design, how do you think mothers / partners will react to being asked to participate in the FERN RCT?
6.2 How long do you think mothers / partners will need to consider trial participation?
6.3 What timeframe (post screening) do you think we should approach mothers / birth partners about trial participation? *Explore: minimum and maximum time frames*
6.4 Would you have any concerns about discussing this RCT with mothers and partners?

Section 7. Training and Resources for the Proposed FERN RCT

7.1 Is there anything specific that you would suggest we include in the FERN site training package?
7.2 Do you envisage any potential barriers to training staff for the proposed FERN RCT?
7.3 Are there any particular resources or other support that you would need to deliver the proposed FERN RCT at your site?

Section 8: Outcomes

Ask participants to refer to the list of outcomes sent prior to the interview.
8.1 Are there any outcomes that you think are important that are not included in the list?
8.2 What do you think would be an appropriate primary outcome for the proposed FERN RCT? *Explore: reasons and alternatives*
8.3 What secondary outcomes would you suggest we measure?
8.4 Considering the outcomes we have just discussed what order would you rank them in, starting with the most important outcomes to measure for this trial and then working down.

Section 9: Overall acceptability

9.1 Overall how acceptable do you think it is to conduct a randomised controlled trial (RCT) exploring active intervention and expectant management for sFGR in MC twins?
Explore answers and rationale
9.2 How do you think you would feel if you were involved in this trial?
Explore: any concerns about decisions about active intervention or expectant management being taken away from them as an individual.
5.7 Do you think a trial exploring active intervention and expectant management is practically possible to conduct? Yes / no *Explore: reasons*, Prompt: logistics and potential solutions

Section 10: Anything Additional

Before we finish, is there anything you think is important for us to know if we conducted the proposed FERN RCT which we have not already covered?
