# Supplementary material for: Balancing key stakeholder priorities and ethical principles to design a trial comparing intervention or expectant management for early-onset selective fetal growth restriction in monochorionic twin pregnancy: FERN qualitative study
Source: BMJ Open. 2024 Aug 9;14(8):e080488. doi: 10.1136/bmjopen-2023-080488 (PMC11331883; doi:10.1136/bmjopen-2023-080488)
Supplement: online supplemental file 7 [file bmjopen-14-8-s007.pdf]

THANK YOU FOR TAKING THE TIME TO READ AND CONSIDER THE DRAFT PROPOSED RCT INFORMATION SHEET. TO BE USED IN CONJUNCTION WITH THE INTERVIEW TOPIC GUIDE PARENT VERSION 1.0 26082020

(To be printed on Hospital Trust headed paper)

Participant Information Sheet

FERN: Intervention or Expectant Management for Early Onset Selective Fetal Growth Restriction in Monochorionic Twin Pregnancy

We are inviting women pregnant with twins to take part in a research study. Before you decide whether or not to take part it is important for you to understand why the research is being performed and what it will involve. Please take the time to read the following information carefully and discuss it with others if you wish. A member of our research team will go through the information sheet with you and answer any questions you may have. Please take time to decide whether or not you wish to take part.

Thank you for reading this.

Why are we doing the study?

The UK has approximately 11,000 twin pregnancies per year with a third of these sharing a placenta (monochorionic (MC) twins). MC twin pregnancy presents extra risks to both the mother and the babies, with some babies dying during pregnancy or shortly after birth. Often this is due to a complication called selective Fetal Growth Restriction (sFGR), where one twin is smaller than the other. sFGR affects one in seven MC twin pregnancies in the UK although we know less about pregnancies where this happens early (before 24 weeks).

There are three main ways of managing MC twin pregnancies with sFGR: 1) a watch and wait approach (also called expectant management), 2) a procedure that blocks the umbilical cord from the smaller twin to the placenta and causes the loss of the smaller twin (also known as selective termination), and 3) a laser that can be used to completely separate the twins' circulations. All of which present significant risks (death and severe disability) to one or both twins.

At present there is a lack of evidence to tell us the best way of managing sFGR in MC twin pregnancies. Currently, women and their partners are offered different management options depending on where they live and who they see. It is also clear that there are gaps in what we know about sFGR.

To be able to find the best way to manage these pregnancies there is much need for a clinical trial comparing management options.

Why have I been chosen?

We are inviting all women aged 18 years and older who are currently pregnant (16 - 23 weeks) with an MC twin pregnancy complicated by sFGR.

Participant Information Sheet

Do I have to take part?

It is up to you to decide whether or not to take part in this study. If you decide to take part - you will be asked to sign a consent form. You will be free to withdraw from the study at any time, without giving a reason. If you decide not to take part – this will not affect the care you or your family receives in any way.

What will happen to me if I take part?

There are three main ways of managing MC twin pregnancies with sFGR: 1) a watch and wait approach (also called expectant management), 2) a procedure that blocks the umbilical cord from the smaller twin to the placenta and causes the loss of the smaller twin (also known as selective termination), and 3) a laser that can be used to completely separate the twins' circulations. All of which present significant risks (death and severe disability) to one or both twins.

If you are currently pregnant and you agree to take part in the study we will use a computer to decide at random which management option 1), 2) or 3) is followed.

1) Expectant: close monitoring but no active intervention. This carries a risk of death of the smaller twin. Death of the smaller twin may result in demise of the larger twin (40%) or disability (30%).
2) Selective termination of the smaller twin. This may protect the larger baby from harm if the smaller twin were to subsequently die. However, termination may not be acceptable to some parents.
3) Selective placental laser photocoagulation of connecting vessels. This is likely to be a complex surgery where Laser is used to close the connections between the babies in the placenta. It may worsen outcomes for the smaller twin.

Will my taking part in this study be kept confidential?

Yes. We will follow ethical and legal practice and all information collected about you and your babies will be handled in confidence. Any information you provide will only be looked at by the research team and will be stored securely. Your information will be coded, and no personal data will be available to the researchers. With your consent, your GP will be notified of your participation in the study.

(To be printed on Hospital Trust headed paper)

Participant Information Sheet

How will you use my data?

How will you use information about me?
We (study sponsor – <<insert Sponsor name>> will need to use information from you and from your medical records for this research project.

This information will include your initials, date of birth, NHS number, name, contact details (telephone number and email address), and the first part of your postcode. People will use this information to do the research or to check your records to make sure that the research is being done properly.

People who do not need to know who you are will not be able to see your name or contact details. Your data will have a code number instead.

We will keep all information about you safe and secure.

Once we have finished the study, we will keep some of the data so we can check the results. We will write our reports in a way that no one can work out that you took part in the study.

What are my choices about how my information is used?

- You can stop being part of the study at any time, without giving a reason, but we will keep information about you that we already have.
- We need to manage your records in specific ways for the research to be reliable. This means that we won't be able to let you see or change the data we hold about you.
- If you agree to take part in this study, you will have the option to take part in future research using your data saved from this study.

Where can I find out more about how my information is used?
You can find out more about how we use your information

- at www.hra.nhs.uk/information-about-patients/
- our leaflet available from www.hra.nhs.uk/patientdataandresearch
- by asking one of the research team
- by sending an email to legalservices@liverpool.ac.uk, or
- by ringing us on 0151 795 0523.

Participant Information Sheet

What are the possible benefits of taking part?

The results of this study will benefit women for counselling as to which management option is best for the smaller and larger twin; both short-term (such as risk of death during pregnancy or in the neonatal period or risks of prematurity) or long-term (such as the risk of disability in the surviving babies).

Taking part will also result in a better understanding of the outcomes for sFGR in MC twin pregnancies managed in a variety of ways and will benefit women in terms of counselling as to which pregnancy management option to choose in the future.

The ultimate goal of this study is to establish the best possible way to manage MC twin pregnancies complicated by sFGR.

What are the possible risks of taking part?

Taking part in this study presents a possible risk to the babies mainly (risk of death whether during pregnancy or the neonatal period, risk of prematurity including the risk of disability) and the mother (likely to be small and related to the surgery, e.g. fetoscopic Laser surgery or selective termination of the smaller twin). Surgical risks include risk of bleeding, infection or injury to internal organs, but as the surgery is usually performed using minimally invasive techniques, the risks are likely to be small.

All the information you provide to us will be collected, stored and used in compliance with data protection regulations (GDPR) and the study will be conducted in accordance with ethical and legal practices.

What will happen if I don't want to continue in the study?

You are free to withdraw from the study at any time, without explanation. The care you or your family receives will not be affected in any way. If you withdraw from the study we will not collect any further information from you. We will however keep and use any information you have already provided.

What will happen to the results of the research study?

It is intended that once the study is complete the results will be publication in peer reviewed journals, presented in national and international scientific meetings and shared with pregnant women through social platforms and the Twins Trust website.

DRAFT

Participant Information Sheet

Where can I get further information or discuss any problems?

If you have any questions or concerns about any aspect of this study, please contact a member of the research team on <<insert telephone number>>. If your concerns are not resolved, you can contact the Patient Advisory Liaison Services (PALS) on <<insert telephone number>>. You can also visit PALS by asking at your hospital reception. If you should need additional support to help with any distress arising from your pregnancy you can contact either the Twins Trust (<<insert email address / telephone number>>) or your GP.

Who is organising and funding the research?

<<insert Funder name>> is funding this study and Professor Asma Khalil is the study Chief Investigator. The study is sponsored by <<insert Sponsor name>> and is managed by the <<insert Research Centre name>>.

Who has reviewed the study?

All research in the NHS is looked at by an independent group of people, called a Research Ethics Committee, to protect your interests. This study has been reviewed for ethical considerations and given a favourable opinion by members of the <<insert REC name>> Research Ethics Committee.

Contact for further information.

Should you have any further queries regarding this study, please contact:

Professor Asma Khalil, Chief Investigator <<insert contact details>>

DRAFT
