# Supplementary material for: Balancing key stakeholder priorities and ethical principles to design a trial comparing intervention or expectant management for early-onset selective fetal growth restriction in monochorionic twin pregnancy: FERN qualitative study
Source: BMJ Open. 2024 Aug 9;14(8):e080488. doi: 10.1136/bmjopen-2023-080488 (PMC11331883; doi:10.1136/bmjopen-2023-080488)
Supplement: online supplemental file 8 [file bmjopen-14-8-s008.pdf]

FERN: Outcomes

THIS INFORMATION WILL BE EXPLAINED TO YOU FULLY DURING YOUR INTERVIEW

- An outcome measure refers to **‘what’** should be measured in a research study to find out whether a treatment is effective.

- Studies often have a number of outcome measures to determine whether a treatment is effective.

- Researchers or doctors often suggest what outcomes should be measured in a research study. However, they do not always fully understand what it’s like to be the mother or partner of a mother who has a problem with their pregnancy. That is why it’s important we ask parents/guardians what outcomes they think a research study should measure to determine whether a treatment is effective.

- Below is a list of outcomes that might be useful to measure. During your interview, we will ask you what you think about the outcome measures on this list.

- It’s not a test! We just want to make sure we include outcomes that are important to parents and children.

- Live birth (baby is breathing or shows any other evidence of life such as beating of the heart, voluntary muscles are moving, and umbilical cord is pulsating at birth)
- Gestational age at birth (the period of time between conception and birth)
- Birth weight
- Intertwin birth-weight discordance
- Death of surviving twin after death of cotwin
- Loss **during** pregnancy **or before** final hospital discharge (miscarriage, stillbirth, termination of pregnancy, neonatal death, perinatal death)
- Parental stress
- Procedure-related adverse outcome (failure of procedure, procedure-to-delivery interval, placental abruption, life-threatening haemorrhage, sepsis, maternal death)
- Length of stay in hospital (neonatal)
- Neurodevelopment impairment / Cognitive ability (how your baby's brain develops and functions)
- Childhood disability (for example, growth, breathing, hearing, visual and gross motor impairments, activity limitations and participation restrictions)
- Child quality of life
- Parent quality of life
