# Supplementary material for: Balancing key stakeholder priorities and ethical principles to design a trial comparing intervention or expectant management for early-onset selective fetal growth restriction in monochorionic twin pregnancy: FERN qualitative study
Source: BMJ Open. 2024 Aug 9;14(8):e080488. doi: 10.1136/bmjopen-2023-080488 (PMC11331883; doi:10.1136/bmjopen-2023-080488)
Supplement: online supplemental file 9 [file bmjopen-14-8-s009.pdf]

# Inclusion and exclusion criteria

**Inclusion Criteria:**

- Monochorionic diamniotic twin pregnancy
- Diagnosis of sFGR (estimated fetal weight (EFW) of one twin <10th centile + EFW discordance >25%)
- Gestational age at diagnosis between 16+0 - 23+6 weeks based on ultrasound
- Informed consent given by the participant and consent form completed and signed

**Exclusion Criteria:**

- Singleton pregnancies
- Maternal age under 18 years
- Other MC complications; twin to twin transfusion syndrome (TTTS), twin anaemia polycythaemia sequence (before enrolment), other rare complicated MC twin pregnancies, such as twin reversed arterial perfusion syndrome
- Known karyotype abnormality at enrolment
- Known major fetal structural abnormality at enrolment, defined as a lethal, incurable or curable severe abnormality with a high risk of residual handicap
- Indication for immediate delivery
- Pre-term pre-labour rupture of membranes before enrolment
- Women who lack the capacity to give informed consent
- Any medical or psychiatric condition which compromises the woman's ability to participate
