# Supplementary material for: Balancing key stakeholder priorities and ethical principles to design a trial comparing intervention or expectant management for early-onset selective fetal growth restriction in monochorionic twin pregnancy: FERN qualitative study
Source: BMJ Open. 2024 Aug 9;14(8):e080488. doi: 10.1136/bmjopen-2023-080488 (PMC11331883; doi:10.1136/bmjopen-2023-080488)
Supplement: online supplemental file 10 [file bmjopen-14-8-s010.pdf]

Clinician suggestions for additions or amendments to the proposed FERN inclusion and exclusion criteria

**Clinician suggestions for additions or amendments to the proposed inclusion criteria**		
**Inclusion criteria should include:**	**Women with Type II sFGR only**	*'I wouldn't take part in it for Type I'* (C3, Doctor).<br>*'I wouldn't agree with offering them intervention if they were Type I sFGR'* (C8, Doctor).<br>*'Expectant management is typically the best option for Type I sFGR. Intervention (in the form of selective termination) is typically the best for Type II sFGR, and … is typically the best for Type III sFGR'* (C2, Doctor).<br>*'Type I would never be an indication for doing a cord coagulation because it's a good prognosis, as long as you have positive flow in the umbilical artery of the smaller foetus. But on the other hand, in Type II, it's quite well predictable when there is foetal deterioration, because you can do it via extensive monitoring, and you look at the foetal circulation… Certainly, Type II would be the one which would qualify most or is most convincing if you decide to do cord coagulation… But if there is persistent reverse flow in the umbilical artery, or zero flow from a very early stage onwards, then the situation is different. It's a very high risk that the smaller baby will die at a certain point. And that's what you want to… So uncontrolled death, let's say, that's one you would like to avoid by doing the study, because the theory would be, or the hypothesis, that it's better to do it in a controlled way by cord coagulation. It's better for the surviving, for the second twin, the normally growing twin. And, at the end of the day, you accept the loss of the smaller twin for the sake of the bigger twin'* (C13, Doctor).
	**Women with an abnormal ductus venosus Doppler** (and carefully consider how the timing of diagnosis can impact the outcome):	*'For the ones where I'm pretty sure that the baby is going to die, that's your ductus venosus A wave absent or reversed, I think it's right that the parents should be able to choose in those situations'* (C8, Doctor).<br>*'I would be stricter on the degree of Doppler abnormalities in the smaller baby regarding ___and umbilical artery and the timing of onset. Yeah, like when there's a big difference when you first diagnose at 23^{+6} weeks or whether it's already there at 16 weeks. So, we know that the earlier, the worse the outcome'* (C2, Doctor).<br>*'If you put … ductus venosus, DV Doppler. This is a severity criteria but actually the children with abnormal ductus venosus are at the highest risk'* (C10, Doctor).
**Amend these inclusion criteria:**	**Estimated fetal weight of one twin to be less than the 3rd Centile** (rather than less than the 10th centile):	*'Move… to less than the third centile… to catch the more severe cases'* (C8, Doctor).<br>*'Maybe you should say, "One twin below the third centile," because then you are sure that it is really a tiny one, or making the estimated foetal weight [discordance] bigger'* (C14, Doctor).

	**Estimated fetal weight discordance to be more than 40%** (not more than 25%):	*'I think the other thing that they should consider adding in the inclusion criteria is the degree of growth discordance, because I think that is where the real uncertainty is. So, for example, if you've got, at 18 weeks, a 40% growth discordance, then it is more likely to deteriorate, the Dopplers are more likely to deteriorate quicker. So, maybe specifying the degree of growth discordance, and maybe more than 40% or more, yeah, at that gestation, because I think there is a dilemma there in terms of clinical management'* (C8, Doctor).<br><br>*'If it's a severe Type II, then the discordance doesn't matter. Like, in my opinion, I think it's not so much the discordance that matters. I think you can easily take also more than a 20% difference in estimated foetal weight. It's more the Dopplers that matter. So, I would be less strict on the estimated foetal weight'* (C2, Doctor).
	**Split estimated fetal weight/weight discordance into two groups – those with mild and those with severe.**	*'The main problem I foresee: that in the current inclusion criteria a milder and a very severe group are merged together. If you want to offer laser, I would say that you should offer that, but it is an interesting thing to offer in the milder group'* (C14, Doctor).
	The **gestational age at diagnosis should be between 18^{+0} [or 20]** (not 16^{+0}) **and 23^{+6} [or 27^{+6}] weeks** based on ultrasound:	To account for abnormal Dopplers that you can get at 16 weeks *'just because of an early gestation effect'* and *'to try and maybe rule out and try and be more specific that we've got the right group of patients and growth-restricted foetuses with Type II selective IUGR'* (C8, Doctor).<br><br>*'I would advocate [the inclusion criteria to be] at least … 20 weeks… and second measurement… [and] a little bit more focused to slightly more severe [sFGR]* (C14, Doctor).<br><br>And *'maybe with the chance to recruit right up to 27^{+6}'* (C11, Midwife).
**Clinician suggestions for additions to the proposed exclusion criteria/Defining current proposed exclusion criteria carefully**		
**Exclusion criteria should include**	**Diagnosis of Type I sFGR**	*'I think most Type I and Type III cases do well without any treatment. So, yeah, I think the criteria are not… These are not severe enough'* (C2, Doctor).<br><br>*'Definitely Type I, because I really don't think it's fair to offer those women that can actually… Where the pregnancies can go on for weeks and weeks, to offer them an intervention that puts them at risk of miscarriage. I think we'll be doing harm there. So, I think Type I should be an exclusion criterion… I certainly wouldn't be offering intervention for Type I IUGR"* (C8, Doctor).

		'It might be difficult to offer it if it is Type I was normal Doppler in a smaller baby' (C1, Doctor).<br><br>*'Type I would never be an indication for doing a cord coagulation because it's a good prognosis, as long as you have positive flow in the umbilical artery of the smaller foetus… so I would never randomise to cord occlusion… because they have a good prognosis'* (C13, Doctor).
	**Diagnosis of Type III sFGR**	*'I think most Type I and Type III cases do well without any treatment. So, yeah, I think the criteria are not… These are not severe enough'* (C2, Doctor).<br><br>*'I've looked after a number of Type III's, and I find that I can usually take both babies to 28 weeks, 26 to 28, where they're both viable… the chances of survival are very good at 28 weeks, but I've monitored them like a hawk… I've not lost a single baby earlier than that, that I've looked at for type three. So, with my own experience with Type III, I wouldn't offer it for Type III'* (C8, Doctor).<br><br>*'And it's similar with class [Type] III, because our experience also has been described in longitudinal observational studies, that they had a very good chance to get on until 30/32 weeks, because they have this arterial anastomosis, which seems to be good for them, let's say it like that. And therefore, I would be hesitant to randomise them'* (C13, Doctor).
	**Bleeding in pregnancy**	*'Bleeding in pregnancy, so bleeding increases the risk of … miscarriage, and I'm not sure it's fair to put those patients through that'* (C8, Doctor).
	**Women with a BMI of over 40**	*'I suppose if you were doing a bipolar cord occlusion or an RFA it is- I don't know. I don't do those, so I don't know how technically difficult it is. But I don't know whether a BMI over 40 would make it tricky'* (C3, Doctor).
**Define these exclusion criteria more carefully:**	**Twin to twin transfusion syndrome (TTTS)**	*'We have found clinically often there can be a bit of a combination of both'* TTTS and sFGR (C3, Doctor).<br><br>*'Sometimes it is a little bit of a grey area between twin-to-twin and selective IUGR. So, … the two conditions may concur concurrently. I think about a third of selective IUGR babies, twins, also have*

		superimposed twin-to-twin transfusion' (C7, Doctor).
	**Known karyotype abnormality at enrolment**	*'I think with monochorionic twins, selective foetal growth restriction is more likely to be secondary to placental problems, rather than karyotypic abnormality. So, I wouldn't make an amniocentesis or a karyotype a requirement'* (C8, Doctor).
