# Supplementary material for: Balancing key stakeholder priorities and ethical principles to design a trial comparing intervention or expectant management for early-onset selective fetal growth restriction in monochorionic twin pregnancy: FERN qualitative study
Source: BMJ Open. 2024 Aug 9;14(8):e080488. doi: 10.1136/bmjopen-2023-080488 (PMC11331883; doi:10.1136/bmjopen-2023-080488)
Supplement: online supplemental file 11 [file bmjopen-14-8-s011.pdf]

Application of FERN qualitative (WP2) findings to the Principles of Biomedical Ethics (Beauchamp and Childress, 2019)

Biomedical ethics principle	Clinician views	Parent views
**Respect for Patient Autonomy** - Supporting parents' decision making and giving parents the freedom to choose how their pregnancy is managed	Although clinicians might support parents with their decision making, their background and culture can impact parent autonomy. Parents find it ethically challenging to terminate the life of one or both their twins.<br><br>*'I think ethically, if the women are consenting to that and are fully informed, I don't think there's too much of a problem there ethically, because the pregnancies are very, very high risk. And I think as long as the women are fully informed and have had a big discussion with the clinician, which they will do, then I think that's probably okay'* (C5, midwife) – but the trouble is 'Veracity' because there is a paucity of evidence available about outcomes for Type I and Type III sFGR.<br><br>*'I think including a third arm of selective termination would exclude a lot of people, I think they wouldn't necessarily take part if that was one of the arms. That arm of selective termination may be the barrier to some people signing up to the trial… termination is not an option for… Asian or ethnic minority population'* (C7, doctor) or parents from Ireland: '*the culture … [and] general consensus … of the population [area or country] and how they see things'* (C10, doctor).<br><br>*'But I think culturally women are still quite- And I am sure it will change with time, but I know if we offer feticide to women with fetal abnormality a lot of them just will continue the pregnancy rather than them having the feticide procedure'* (C3, doctor).<br><br>Parents *'struggle with … the decision to actually actively choose to terminate the life of one baby… [even] when trying to save the life and optimise the outcome of the other baby'* (C8, doctor).	Parents felt (or were told by clinicians) that they were unequipped to make decisions about which pregnancy management route to take. Parent were not always given all the management options. Although parents would have the choice in whether to participate in the RCT or not, they would be randomised to a pregnancy management option which they would not choose and which goes against autonomy. Parents can feel pushed into making decisions within the legal timeframe for termination:<br><br>*'If they are given an option, every woman has the right to decide which option they take. I just feel, for me, that the prerequisite or the criteria probably should be thought about a little bit more. It should be based on individual cases, how you select it, as opposed to randomly, any woman that has this diagnosis'* (P19, mother, site).<br><br>*'We went back into the hospital and then I think they had realised that, probably because we had been gone so long, I don't know, that maybe we were not equipped to make this decision… 'She had opportunity to say, "Actually I think this is the wrong course" [expectant management]. Because they said, "You can change your mind. You've got up until 26 weeks to change your mind." We still had a few weeks, even though I think we probably wouldn't have wanted to push it. I don't know'* (P2, bereaved mother, social media).<br><br>*'At that point the consultant was like, "We're going to run out of time for you to have anything done. If you do want to do selective termination, you need to do that now'* (P11, mother, social media).<br><br>*'One of those options is choosing to have an abortion, which I know a lot of people feel very strongly about. I don't think I'd participate. I'd rather feel like I was getting the balanced view*

		from your doctors and your consultants about what they think will be the best option for you and your individual circumstances, rather than it being random' (P3, mother, currently pregnant, site).<br><br>*'I think one of the options they also gave us was, "You don't have to come in and have scans, you can just say goodbye and not have a conversation with us again until birth." They obviously, at that point, said, "We don't recommend that one in particular," but that was still given as an option, of, "You don't have to do any of this that we're talking about. It's your body, your children, etc., you can do what you would like to do"'* (P14, partner, social media).
**Beneficence** - Doing their best to save the lives of one or both babies	Clinicians indicated that they wanted to save the lives of at least one, but preferably both babies – Live birth was the top ranked outcome for clinicians and parents. The proposed trial will answer an important research question to guide clinical practice and discussions with parents*:*<br><br>*'I have had patients come to me who were offered a selective termination within somebody from the team, and I felt that it was a different type of selective IUGR. So, somebody thought it was Type II but I thought it was Type III, and I would manage Type III differently… Somebody in the team thought it was Type II, which deteriorates much faster and in a predictable way. And Type III can go on for a long, long time, but it's an unpredictable, sudden loss. So, I have scanned a patient like that, where I was asked for a second opinion, and I said that, "This is Type III and I wouldn't offer a termination." I wouldn't offer a termination… And we took them to 26-plus weeks, and they delivered the twins, both alive, and I get pictures from them still, and they're so grateful. And they do say that, that, "Oh, we were offered a termination, but actually, you said that it was okay and we just carry on. And we're so grateful to you that both our babies are here today""'* (C8, doctor).	Parents wanted to save the lives of both babies – Live birth was the top ranked outcome for parents and clinicians.<br><br>'*I knew that if we intervened Twin 2 would almost certainly die. But we did decide, they seemed to think he wasn't going to survive anyway so we sort of came to the decision that we had a responsibility to Twin 1 to do what was best for him*' (P2, bereaved mother, social media).<br><br>*'I think I would need statistics to show me that it was better for the bigger baby, that their chances of being born healthy were significantly better if the cord occlusion took place. I think because my smaller twin was always really healthy, that would've always been a no from us. But had our smallest twin shown signs of being poorly or showing signs of not being compatible with life once they were born, that would've made a difference'* (P4, mother, social media).
**Justice** - Supporting parents and their babies' legal rights, allocating resources,	Selective termination of smaller twin does not support that twin's legal right. There is not enough evidence to show the risks of intervention to the larger twin:	'*By sacrificing those babies who might have survived in order to increase the chance of future babies surviving it feels wrong to me'* (P18, partner, site).

equal respect, non-discrimination	'*Most centres would go for cord coagulation of the smaller twin. But, on the other hand, that means that, by default, by definition, this means mortality for this pregnancy of 50%. It's foetal mortality, because it's like controlled feticide. And therefore…Well, it would be possible, it is also ethically and legally possible in [European Country], but we also have the experience that there is still a higher than 50% double survival rate in selective foetal growth restriction, if you have conservative monitoring. So why would you do a selective cord coagulation in the first place? The main argument for doing a cord coagulation is that it may prevent damage to the surviving foetus, because you have closed the vascular circulation of that foetus. But, on the other hand, no trial so far has ever shown that the neurological morbidity is lower after cord coagulation of the surviving twins than if you have conservative management, which may include intrauterine foetal death of one of the twins and then the other one is at risk as well*' (C13, doctor).	'*I knew that if we intervened Twin 2 would almost certainly die. But we did decide, they seemed to think he wasn't going to survive anyway so we sort of came to the decision that we had a responsibility to Twin 1 to do what was best for him'* (P2, bereaved mother, social media) – parents ended up going with expectant management, lost both twins.
**Non-Maleficence** - Not harming parents or their babies	Every case needs managing differently. Some clinicians felt strongly that the trial inclusion criteria should only include women with Type II sFGR and women with an abnormal umbilical artery Doppler Types I and III generally do not require intervention.<br><br>A clinician (C8, doctor) recalled that they gave a second opinion on a colleague's recommendation for selective termination and found that the sFGR was type III, and not type II, so recommended expectant management. The family had two healthy children and send photographs to them every year thanking them for not terminating the life of their smaller twin who is healthy (see quote in 'Beneficence').<br><br>Similarly, to reduce harm to parents, discussions regarding the different management options should be tailored to each individual depending on the severity of their pregnancy, because even the mention of selective termination causes long-term harm to parents:<br><br>*'But what you also see, if patients are rational and can cope with these kinds of things, that is a good approach, but you also see, in our hospital, we do a lot of follow-up visits also at the age of four, eight and twelve years old. Then, still, people are telling to the psychologists who do, they also do Bayley Scales testing and that kind of thing, that the mentioning of the option of cord occlusion was one of the worst things*	Evidence of potential long-term harm to parents by them being offered and even considering selective termination of one of their twins. Regret for a bereaved mother for not choosing the selective termination option. Regret for the mother of two healthy twins that they even considered selective termination: *'Did they [parents] survive emotionally… after the decisions… to terminate one of these kids? … I am not going to go and kill myself because I have made the wrong decision? ... How the hell do you, as a mother, cope? I don't want to think about it. Not even close to wanting to think about it. But it is one of those questions you have to ask yourself'* (P16, Mother, site).<br><br>One mother, who had initially decided to take the intervention route but had been told by the consultant to *'do nothing'* and whose twins had both gone on to die in utero at just over 26 weeks, said that *'if there had been conclusive evidence that had said intervention was the better option, I would have done it. I would have gone back the next day and done it'* (P2, bereaved mother, social media). On the other hand, a mother who had Type II sFGR (from which she understood, and from what a clinician also said was the type *'that has the most negative outcomes'* (P9, mother, social media) *'without intervention [when] abnormal ductus venosus'* (C8, doctor) who

	that happened to them in the monitoring of the pregnancy. So, I think, as FMFs, because those people are with the paediatrician, of course, we don't see those patients back again. As FMF, we should take this into account. Those patients who are traumatised by mentioning the option of cord occlusion, they say, they report back, that every time they entered the hospital – and it was sometimes far later – we, as the doctors, already thought that there was a forgotten option, if you understand what I mean, but the patient, the parents themselves, felt that every time they visited the hospital the doctor could say that one of the babies had to die' (C14, doctor).	was not given a choice and was put onto the expectant management route and whose twins were both born and remain healthy and free of any disability at 17 months old said *'if I'd been selected into the cord occlusion [selective termination], it's a [her smaller twin's] life that, potentially, wouldn't need to have been lost there'* (P9, mother, social media); a healthy baby's life would have been ended.
**Fidelity** - Being loyal and providing parents with support throughout their pregnancy and the post-natal period; being worthy of a patient's trust	Clinicians monitor their patients *'like a hawk'* and are *'really invested in them':*<br>*'Type III, I would want to consider that carefully, and I'd probably want to have a discussion with other experts in terms of their experience. But, I'm uncomfortable offering it to Type III's from my own limited experience, and Type III is less common, but where I have looked after the Type III's, I've taken them to 28 weeks and delivered two live babies. And the chances of survival are very good at 28 weeks, but I've monitored them like a hawk. So, it is just, you take ownership of the patient and you become really invested in them, but then, it's very fulfilling and good for them if you can deliver two live babies'* (C8, doctor).	Parents want clinicians to express their views in order to support the patient in making a decision that would give them the best outcome in their situation. Parents also have trust that clinicians will manage the pregnancy according to need:<br><br>'*I think I would've always trusted what a consultant had recommended, potentially with a second opinion. I wouldn't have been against it if that was what would've been best. It's not something I'm completely against if that is what would give us the best outcome, then that's what we would've done'* (P4, mother, social media).
**Veracity** - Being honest and truthful with parents; not presenting parents with misleading information	Clinician data suggests that there is a lack of evidence-based information that can be provided to parents to inform their decision making about trial participation and expectant management versus intervention outcomes. Clinicians gave conflicting advice to women:<br><br>*'I have had patients come to me who were offered a selective termination within somebody from the team, and I felt that it was a different type of selective IUGR. So, somebody thought it was type two but I thought it was type three, and I would manage Type III differently… Somebody in the team thought it was Type II, which deteriorates much faster and in a predictable way. And Type III can go on for a long, long time, but it's an unpredictable, sudden loss. So, I have scanned a patient like that, where I was asked for a second opinion, and I said that, "This is Type III and I wouldn't offer a termination." I wouldn't offer a termination…* (see rest of quote in 'Beneficence')*'* (C8, doctor).	Parents need to be presented with enough information about the study, risk and benefits, and reassurance around the ethics of it.<br><br>*'I guess reassurance that it had been approved as safe research to be doing and ethical. Reassurance about how my information and data would be used. Then, yes, I guess a really clear rationale for why it is needed, especially because it's such a… Not invasive. Invasive is the wrong word. But it's not like an observational study. It could actually affect what happened… What happens to your babies'* (P2, bereaved mother, social media).<br><br>*'What I like about this [proposed trial] information sheet is it actually says things that I never knew. 'Close monitoring, but no active intervention this carries a risk death to the smaller twin. Death to the smaller twin may result in demise of the*

	'The data that we have now… comes from different observational studies with their own risks of bias' (C2, doctor).<br><br>*'I think a trial will be important because I think the literature is very biased'* (C1, doctor).<br><br>*'We don't really have that information … about outcomes… at the minute… and you can't predict that really [for Type I and Type III sFGR]'* (C3, doctor).	*larger twin, 40%...' I didn't know that, that's helpful… That's helpful to actually know that'* (P11, mother, social media).
**Confidentiality and privacy**	Laws on abortion changing but culturally, patients still continue with the pregnancy in some areas where abortion laws were in place until recently:<br><br>*'The abortion laws have changed here. So, we now would offer feticide. But I think culturally women are still quite-'* (C3, doctor).	*'I guess reassurance that it had been approved as safe research to be doing and ethical. Reassurance about how my information and data would be used'* (P2, bereaved mother, social media).
