# Supplementary material for: Balancing key stakeholder priorities and ethical principles to design a trial comparing intervention or expectant management for early-onset selective fetal growth restriction in monochorionic twin pregnancy: FERN qualitative study
Source: BMJ Open. 2024 Aug 9;14(8):e080488. doi: 10.1136/bmjopen-2023-080488 (PMC11331883; doi:10.1136/bmjopen-2023-080488)
Supplement: online supplemental file 12 [file bmjopen-14-8-s012.pdf]

Application of FERN qualitative (WP2) findings to the Adapted Theoretical Framework of Acceptability (Deja et al., 2021*; Sekhon et al., 2018)

Construct & definition	Fully met for parents?	Fully met for clinicians?
**Affective attitude:** *How an individual feels about the intervention.*	**No:** Most parents clearly stated that they would not consent for their child to take part in the FERN RCT. The few that would hypothetically agree to participate would withdraw if not happy with allocated arm.<br><br>*'Why would it be done in that way rather than giving people the balanced options and then finding out the outcomes of each one or has that already been done? ... Overall, I think it is a bit worrying to be randomly choosing someone. That's the ethical side of things, randomly choosing someone to have a certain* loss *would be something I'd be concerned about'* (P3, mother, social media).	**No** Whilst seeing the merit of doing an RCT, most clinicians did not find the proposed FERN RCT acceptable overall and '*wouldn't take part […] We have ethics raised about lots of different studies and we work through them most of the time, but I don't think we could work through this one'* (C11, Doctor).<br><br>RCT acceptable if inclusion criteria changed:<br><br>*'I would say yes, it is acceptable with the confinement to the very severe growth restriction, which we have discussed, which is type II'* (C13, doctor).<br><br>However, this is still problematic if someone requiring intervention is allocated to expectant management.
**Burden:** *The perceived amount of effort that is required to participate in the intervention.*	**No:** Having to decide which pregnancy management option to take was described by parents as traumatic. Adding the option of a study to an already stressful situation may add to the burden:<br><br>*'I think it would be really helpful to not have that couple of hours crying in the car, because we had been presented with these options and had no idea, and no one seemed to have any idea what was the best one to do. That was quite traumatic'* (P2, bereaved mother, social media).<br><br>Moreover, some parents spoke of the added burden of feeling like they *'disappointed'* (P14, partner, social media) the clinician or as if they are *'bad people'* for not participating:<br><br>*'It's removing that guilt away from the parent, I think, as well. If they don't go ahead [with the trial/allocated arm], then firstly, it doesn't make them bad people if they don't want to make that choice. That's alright, you don't have to make that choice [to participate in the study]. You can go with your gut instinct and do what you think is best for you and your babies'* (P12, mother, social media, joint interview).	**Depends** on site and the management options they offer (i.e., patients randomised to intervention at a referral site would need to be referred to a tertiary hospital – hence reducing the burden on the referral hospital who would have instead expectantly managed the pregnancy on site).

**Ethicality:** *The extent to which the intervention has a good fit with an individual's value system*	**No:** Timing of approach, mentioning of selective termination causes harm, personal and cultural views of selective termination<br><br>*'In a way it is contradictory, it is opposite courses, like you want to be ethical but it is almost like an impossible task to be ethical, so it is more like… You can't be ethical basically, I don't think. Am I making sense? It is almost like the mission impossible and you just need to find a way to kind of… There will be damage basically, you can't avoid it, there is no way, there is no other way'* (P18, Partner, site).<br><br>'*Regret on if you were put in the bracket of selective termination, that's never an option for any parent, never, that's not a decision you can… It's not fair really, isn't it? It's borderline abortion in my eyes, I don't think I'd like that'* (P6, Partner, social media).	**No:** Some clinicians had concerns about causing *'harm'* to babies and parents. They felt that pregnancy management should be individualised.<br><br>Clinicians spoke of how their patients' values would make the selective termination arm unacceptable to them:<br><br>*'[Town] has a very high rate of Asian or ethnic minority population, termination is not an option for them'* (C7, doctor).<br><br>*'But I think culturally women are still quite- And I am sure it will change with time, but I know if we offer feticide to women with fetal abnormality a lot of them just will continue the pregnancy rather than them having the feticide procedure'* (C3, doctor).<br><br>'*I would not start a conversation because it is not acceptable for me first of all, and I don't think it is acceptable for anybody, as I said not ethical to be randomised to kill your own child. So, just imagine to ask a computer to decide for you which child will survive. (Laughter). Or which child will be brain damaged, that is not… They [parents] have to decide what they do, so you give them percentages, you give them risk factors in a particular scenario'* (C10, Doctor).
Intervention coherence: *The extent to which the participant understands the intervention and how it works.*	**No:** Clear and understandable trial materials but uncertain about understanding that laser treatment arm is not selective termination or the risks and benefits of either intervention option. If randomised to intervention, parents would drop out (especially for selective termination).<br><br>*'I feel quite conflicted about it. I think it's [the proposed RCT is] really needed and I really, really want there to be research like this that's been done, because I think it would have been really helpful for us. Even if it hadn't led to Twin 1 surviving, I don't know, I think if there is research found that intervention was more effective, had better outcomes and there was good evidence to show that I think we would have chosen to do it, and then Twin 1 might have survived' (*P2, bereaved mother, social media).	**No:** Clinicians understand the intervention and how it works, however, clinicians cannot agree on the risks and benefits of each intervention due to limited evidence.<br><br>*'Well, that's almost impossible to predict because emotionally it is a very difficult situation for the parents. But I think, in that situation, what we just have defined, when we would randomise, you can clearly tell the parents, with all honesty, that there's equipoise. We don't know. We don't know what's better'* (C13, doctor).
**Opportunity costs:** *The extent to which benefits, profits, or*	**No:** Although a few parents would hypothetically consent to their child taking part in the trial for altruistic purposes (to answer the research question and help families in the future), parents would want to go down the pregnancy management route that their clinician recommended and that they felt they had a choice in.	**No:** Clinicians prefer expectant management for types I and III sFGR and intervention (especially selective termination over laser treatment to increase the likelihood of parents taking a baby home) for type II sFGR with abnormal Dopplers. This would mean that, in the context of the proposed trial, they would have to give up their beliefs and values if

values must be given up to engage in the intervention.	Whilst most parents preferred the expectant management arm of the trial, some spoke of how they would find selective termination acceptable only in severe cases and would want to go down that route if that's what the clinician recommended (i.e., if their condition was severe, they wouldn't want to be in the expectant management arm):<br><br>'*I think if I was pregnant with twins again and had the same problem I would take part in a study like this, because I would want to be part of helping there to be better research. But I would not hesitate to withdraw if I felt that I was assigned to an option that I didn't think was going to give us the best outcome for our babies'* (P2, bereaved mother, social media).	their patient was randomised to an arm that was not the best option for that individual pregnancy.<br><br>*'That is why we have expectant management as the default. And, also, from a pathophysiological perspective, you don't expect laser to improve the outcome. And also, if you look at the literature, that's also what comes out of it. So, most of the time you lose the smaller baby and if you're really unlucky, then you lose the bigger baby, and then you have an even bigger problem'* (C2, doctor).<br><br>Clinicians would be selective about who they recruit to the trial. They would not randomise women with type I or III sFGR to selective termination when that family would have a chance to take two healthy babies home because these types generally have more favourable outcomes:<br><br>*'… [But], especially in…the greater growth… [Type] III situations, you are frequently surprised by how well the small ones keep growing'* (C14, doctor).
**Perceived effectiveness:** *The extent to which the intervention is perceived likely to achieve its purpose.*	**No:** Differences in pregnancy management are already happening and practice varies; Timing of approach and mentioning intervention especially sensitive:<br><br>*'I feel like when it hit 24 weeks and I was being asked to make decisions about whether to keep a baby or not, I almost wasn't able to clearly… I felt very separate from my…. almost disassociated from myself. I felt very separate from what was going on just because I'd been so detached. It was a really weird experience. It's funny, even when I gave birth I hadn't really… It wasn't until I saw them that I was like, oh my gosh, I'm having babies'* (P11, mother, social media).	**No:** Clinicians would not be happy to randomise women with Types I and III sFGR to the intervention arm, and similarly would not be happy to randomise women with Type II sFGR with abnormal Dopplers to the expectant management arm. Differences between evidence on the effectiveness of laser or selective termination.<br><br>*'Parents … [are] more likely to take a baby home if they have a selective reduction, compared to if they had a laser'* (C8, doctor).<br><br>'*There's a big difference between laser and cord occlusion… I don't think laser has… that's a little bit the issue that I don't believe in laser as a treatment for selective foetal growth restriction, unless you see that the smaller baby is going to die and the parents do not opt for, or they cannot, mostly because of religious beliefs, go for a selective reduction'* (C2, doctor).<br><br>*'I think it is probably true that the cord occlusion is potentially the option to maximise the chance of having one healthy child, which is maybe potentially avoiding the risk of significant prematurity. However, I think that might be the method for only the cases where maybe there are abnormal Dopplers. Because I think where the Doppler of the smaller*

		baby is normal, I think it is reasonable to offer expectant management' (C1, doctor).
**Self-efficacy:** *The participant's confidence that they can perform the behaviour(s) required to participate in the intervention.*	**No:** Whilst the parent information leaflet provides clear and understandable information, most parents reported that they were under a lot of stress during initial meetings and found it difficult to comprehend the information that was presented to them. *'It was like really top-line understanding of what the next thing could look like, which, in fairness, is probably best because it's so much information to take in initially, anyway, that I probably wouldn't have heard what she was saying, even though I was listening'* (P15, mother, site).	**No:** Most clinicians did not believe that they can perform the behaviours required to participate in the RCT. Clinicians do not think that the FERN trial is practically possible to conduct in a randomised fashion because management of MC twin pregnancy with sFGR requires an individualised approach, due to the many factors that influence their decision making, and parents need to be provided with high quality evidence to inform their decisions, which is currently lacking. *'What I want to say, and tried to say from the beginning, is we cannot randomise them ourselves, they [parents] will decide the management and then we can put them in that category to reach to some conclusions at the end. But we cannot randomise them, so they have to decide what they want to do'* (C10, doctor).
Trust*: *The extent to which the parent / guardian trusts those delivering the intervention to put the needs of patient before the requirements of the study.*	**Yes:** Parents trusted the opinions of clinicians. However, this makes the proposed RCT difficult to carry out if the clinician has strong opinions on management options or if parents receive conflicting information from different clinicians. *'Being really honest, I think we probably would've done [hypothetically consented to take part in the RCT]. But if we'd got the randomised option and we were like, "This doesn't feel right," and our consultant is going, "Oh, oh, oh, oh," we might've dropped out'* (P1, bereaved partner, social media). *'He [consultant] reached out to one guy in Germany and one guy in the USA, and he said that one of them went, "Why on earth would you intervene? There's no proof that this works. Why would you do that?" The other guy went, "Why on earth wouldn't you do it? There's something available to you. Why wouldn't you intervene if you think there's a big problem?'* (P1, bereaved partner, social media).	N/A
