# Supplementary material for: Balancing key stakeholder priorities and ethical principles to design a trial comparing intervention or expectant management for early-onset selective fetal growth restriction in monochorionic twin pregnancy: FERN qualitative study
Source: BMJ Open. 2024 Aug 9;14(8):e080488. doi: 10.1136/bmjopen-2023-080488 (PMC11331883; doi:10.1136/bmjopen-2023-080488)
Supplement: Supplementary file 13 [file bmjopen-2023-080488.reviewer_comments.pdf]

PEER REVIEW HISTORY

BMJ Open publishes all reviews undertaken for accepted manuscripts. Reviewers are asked to complete a checklist review form (**http://bmjopen.bmj.com/site/about/resources/checklist.pdf**) and are provided with free text boxes to elaborate on their assessment. These free text comments are reproduced below.

ARTICLE DETAILS

**TITLE (PROVISIONAL)**	Balancing key stakeholder priorities and ethical principles to design a trial comparing intervention or expectant management for early-onset selective fetal growth restriction in monochorionic twin pregnancy: the FERN qualitative study
**AUTHORS**	Mitchell, Tracy; Popa, Mariana; Ashcroft, Richard; Prasad, Smriti; Sharp, Andrew; Carnforth, Christine; Turner, Mark; Khalil, Asma; Fenwick, Natasha; Leven, Shauna; Woolfall, Kerry

VERSION 1 – REVIEW

**REVIEWER**	Mitchell, Eleanor<br>University of Nottingham Faculty of Medicine and Health Sciences, Nottingham Clinical Trials Unit
**REVIEW RETURNED**	23-Oct-2023

**GENERAL COMMENTS**	Thanks for asking me to review this manuscript which reports a qualitative study about the experiences of parents and clinicians in the management of selected fetal growth restriction, and the acceptability of a randomised trial in this area. The manuscript is well written and a very interesting read. This is clearly a very complex, challenging clinical and ethical area and I feel the paper adds value to the evidence base, particularly for clinicians who may be interested in designing trials in this area in the future. I particularly enjoyed seeing how the themes generated from the research were applied to ethical principles.<br><br>Whilst I wouldn't expect this paper to give lots of detail about the proposed trial design, it would be helpful to add a few lines/short paragraph at the beginning of the methods/study design section to give the reader an indication about the proposed trial parents and clinicians were asking to give their opinion of. This is in addition to the information provided in supplementary material.<br><br>Table 2 – numbers don't add up to the denominator of 14. In some instances, totals are 10, others 15<br><br>PPIE looks strong and involved at all stages of the study – is there a rationale for these individuals not being included in the authorship?<br><br>The conclusion states that an RCT in this area is not acceptable to parents or clinicians and recommends alternative study designs to be used. It would be helpful for the authors to briefly comment on what designs may be appropriate.<br><br>Other minor points:

	- Title would be improved if the methodology was mentioned and the name of the study. Suggest adding the words ‘qualitative’ and FERN study.<br>- Page 3, row 3: states that early-onset sFGR is less common – are you able to quantify this?<br>- Page 3, row 45 (methods): first mention of FERN study. Can you specify this in full and give a web link or reference to information about the wider programme of work?<br>- Page 5, row 14: suggest adding a web address for Twins Trust and a brief sentence to describe the organisation<br>- Page 5, row 22: worth mentioning ‘qualitative’ feasibility studies to be explicit<br>- Table 1 – not clear what ‘intervention’ hospitals are or ‘local / referral’ hospitals are? Is this to do with the proposed RCT design?<br>- Manuscript moves from reporting participant characteristics into the themes reported, would flow better if there was a sentence introducing the themes, rather than going straight into heading of the first theme<br>- Theme “an important question to answer” states that both clinicians and parents felt it is an important question to answer – 3 quotes are included, all from clinicians. Would be good to see a parent quote here either instead of or in addition to quotes currently included.<br>- Spelling error of Principal in the acknowledgments section

**REVIEWER**	Gebb, Juliana<br>The Children's Hospital of Philadelphia
**REVIEW RETURNED**	29-May-2024

**GENERAL COMMENTS**	I applaud the authors on an excellent paper investigating the perspectives of both patients and clinicians on the complex topic of sFGR counseling, management, and potential for a randomized trial. It is clear that a randomized trial is not acceptable and this is important to publish as cohort studies should be accepted as the next best data source to guide management. I do think that it would have been better to exclude pregnancies with Type I sFGR as many clinicians don't consider intervention in these pregnancies. This is mentioned in the discussion but I believe it should also be added as a limitation.

VERSION 1 – AUTHOR RESPONSE

**Reviewer 1**<br>Title would be improved if the methodology was mentioned and the name of the study. Suggest adding the words ‘qualitative’ and FERN study	The methodology and FERN have been added to the title. FERN has also been introduced at the very beginning of the abstract. The title is now: ‘Balancing key stakeholder priorities and ethical principles to design a trial comparing intervention or expectant management for early-onset selective fetal growth restriction in monochorionic twin pregnancy: FERN qualitative study’.<br><br>The first sentence of the abstract reads: Objectives: ‘As part of the FERN feasibility study, this qualitative research aimed to explore

	parents' and clinicians' views on the acceptability, feasibility, and design of a randomised controlled trial (RCT) of active intervention versus expectant management in monochorionic (MC) diamniotic twin pregnancies with early-onset (prior to 24 weeks) selective fetal growth restriction (sFGR).
**Editor**<br>Please ensure that each point of the Strengths and Limitations section (after the abstract) is no longer than a sentence.	The following sentences have been removed from the limitations section that follows the abstract so that the limitation is now no longer than a sentence:<br><br>Limited to participants who could speak English. Considering the topic, culture and religion may play a factor when making these decisions. The views, experiences, and understanding of the decision parents are asked to make should be explored with families that might have this information presented to them via an interpreter.
**Reviewer 1**<br>PPIE looks strong and involved at all stages of the study – is there a rationale for these individuals not being included in the authorship?	Natasha Fenwick and Shauna Leven have now been added to the author list as they met the International Committee of Medical Journal Editors' (ICMJE) criteria for authorship.<br>The wider PPIE group contributed at different stages so are named in the Acknowledgements Section of the paper as per their permissions and the International Committee of Medical Journal Editors' (ICMJE) criteria for authorship.<br><br>The author contributions section has now been updated to include Natasha Fenwick and Shauna Leven (and their affiliations, contact details and Orcid numbers included on the BMJ Open system, and conflict of interest disclosures completed).
**Introduction**	
**Reviewer 1**<br>Page 3, row 3: states that early-onset sFGR is less common – are you able to quantify this?	Now quantified, with the sentence now reading as: One study investigating 119 pregnancies (n=75/63% early onset sFGR and n=44/37% late onset sFGR) showed that in early-onset sFGR, survival of one (n=62/82.7%) or both twins (n=55/73.3%) were lower compared to late-onset sFGR (one twin n=42/95.5%; both twins n=39/88.6%) [8].
**Methods**	
**Reviewer 1**<br>Page 3, row 45 (methods): first mention of FERN study. Can you specify this in full and give a web link or reference to information about the wider programme of work?	The trial protocol is currently under review but reference 9 is a link to the study webpage and reference 10 is a newly accepted paper for the international survey, so the sentence now reads as: The FERN study [9] involved three work packages, including 1) prospective UK

	multicentre observational study, 2) qualitative study, 3) international survey [10] and consensus meeting. This paper presents the findings of the qualitative work package 2 phase of the study.
**Reviewer 1**<br>Would be helpful to add a few lines/short paragraph at the beginning of the methods/study design section to give the reader an indication about the proposed trial parents and clinicians were asking to give their opinion of. This is in addition to the information provided in supplementary material.	The above paragraph then continues to give a brief indication about the proposed trial parents and clinicians were asking to give their opinion of:<br><br>The aim of work package 2 was to explore parents' and clinicians' perspectives on how the future clinical trial should be designed (including recruitment and consent approaches and the design of research materials), the factors that influence parents' and clinicians' decision making, and the acceptability of a future clinical trial.<br><br>For clarity, we have also added: Following ethics approval (REC reference: 20/SW/0156), we conducted online or telephone interviews with English-speaking women and their partners (where applicable) in the United Kingdom (UK) who were experiencing early-onset sFGR in MC twin pregnancy (or with experience in the last three years), and English-speaking clinical staff involved in the management of MC twin pregnancies in the UK and Europe, to explore their views on the feasibility, acceptability and design of a proposed RCT, with one 'watch and wait' expectant management arm, and two intervention arms: 1) selective termination of the sFGR twin, and 2) laser treatment, for early-onset sFGR in MC twin pregnancy…
**Reviewer 1**<br>Page 5, row 14: suggest adding a web address for Twins Trust and a brief sentence to describe the organisation	The Patient and Public Involvement and Engagement (PPIE) section now reads as:<br>Our PPIE members include six co-applicants: Michelle Watson, Jessica Mendoza, Danielle Harding, and Joel Marsden (two with personal experience of sFGR in MC twin pregnancy) and Natasha Fenwick and Shauna Leven from Twins Trust (https://twinstrust.org), which is a registered charity who support parents through every milestone of their journey with twins, triplets or more. The PPIE members were involved in the grant development, design, recruitment for, conduct, progress and/or findings of the FERN study; and/or as members of the study oversight/steering committee; and/or attended the work package 3 Key Stakeholder meeting in London (3rd July 2023); and/or reviewing and providing input on

	draft research information materials for this qualitative study and reviewing drafts of this manuscript.
**Reviewer 1**<br>Page 5, row 22: worth mentioning ‘qualitative’ feasibility studies to be explicit	Added. The sentence now reads as: ‘Based on previous qualitative feasibility studies [11, 14] we…’
**Findings**	
**Reviewer 1**<br>Table 1 – not clear what ‘intervention’ hospitals are or ‘local / referral’ hospitals are? Is this to do with the proposed RCT design?	This has now been made clear in the Parent Characteristics Table (1):<br><br>Intervention sites (sites that perform selective termination or fetoscopic laser treatment for sFGR) (*n*=8)<br><br>Local/Referral sites (sites who do not perform selective termination or fetoscopic laser treatment for sFGR and refer to the above hospital sites) (*n*=5)
**Reviewer 1**<br>Numbers don’t add up to the denominator of 14 [in the Clinician Characteristics Table]. In some instances, totals are 10, others 15	Thanks so much for noticing this! The numbers have now been updated to be correct.
**Reviewer 1**<br>Manuscript moves from reporting participant characteristics into the themes reported, would flow better if there was a sentence introducing the themes, rather than going straight into heading of the first theme	The following sentence has now been added:<br><br>Six interlinked themes will now be presented, which highlight the importance that parents and clinicians place on answering the research question and considering the practical and ethical challenges of conducting the proposed clinical trial.
**Reviewer 1**<br>Theme “an important question to answer” states that both clinicians and parents felt it is an important question to answer – 3 quotes are included, all from clinicians. Would be good to see a parent quote here either instead of or in addition to quotes currently included	*The clinician quote: ‘I think clinicians just need guidance on what to do depending on the stage’’* (C11, midwife) has been replaced with a parent quote: *‘I think it’s great that you’re doing something to help parents make decisions… there wasn’t really a lot of information… that me and my wife could find’* (P14, partner, social media).
**Conclusion**	
**Reviewer 1**<br>The conclusion states that an RCT in this area is not acceptable to parents or clinicians and recommends alternative study designs to be used. It would be helpful for the authors to briefly comment on what designs may be appropriate.	Drawing on findings from the wider FERN study, as well as the barriers identified in this study to both recruitment and retention, alternative study designs such as an international multicentre observational cohort study or propensity score matching should be considered to address this important research question.
**Strengths and limitations of the study**	
**Reviewer 2**<br>I applaud the authors on an excellent paper investigating the perspectives of both patients and clinicians on the complex topic of sFGR	As part of the feasibility study, it was important to consider views on the inclusion and exclusion criteria for the proposed trial. We have not added this as a limitation of the study as the

counseling, management, and potential for a randomized trial. It is clear that a randomized trial is not acceptable and this is important to publish as cohort studies should be accepted as the next best data source to guide management. I do think that it would have been better to exclude pregnancies with Type I sFGR as many clinicians don't consider intervention in these pregnancies. This is mentioned in the discussion but I believe it should also be added as a limitation.<br><br>**Editor**<br>Please ensure that each point of the Strengths and Limitations section (after the abstract) is no longer than a sentence.	exclusion of Type I sFGR was a finding in this feasibility study rather than a limitation of the proposed clinical trial.<br><br>The second sentence that was removed from the Strengths and Limitations section (after the abstract) has now been placed in the strengths and limitations section that follows the conclusion:<br>The views, experiences, and understanding of the decision-making parents are asked to make should be explored with families via an interpreter.
**Acknowledgements**	
**Reviewer 1**<br>Spelling error of Principal in the acknowledgments section	Thank you for noticing this. This spelling error has now been corrected.

We attach a clean and a marked copy (using track change function) of our revised article.

Thank you, once again, for all your work in considering and reviewing our article.

Kind regards,

Dr Tracy Mitchell and the FERN study team
1

VERSION 2 – REVIEW

**REVIEWER**	Mitchell, Eleanor<br>University of Nottingham Faculty of Medicine and Health Sciences, Nottingham Clinical Trials Unit
**REVIEW RETURNED**	08-Jul-2024

**GENERAL COMMENTS**	Thank you for asking me to review this revised manuscript. The authors have now addressed all points and I believe the manuscript is acceptable for publication.
